# Wastewater Treatment by Polymeric Microspheres: A Review

**DOI:** 10.3390/polym14091890

**Published:** 2022-05-05

**Authors:** Jiwon Lee, Rajkumar Patel

**Affiliations:** Energy and Environmental Science and Engineering (EESE), Integrated Science and Engineering Division (ISED), Underwood International College, Yonsei University, 85 Songdogwahak-ro, Yeonsu-gu, Incheon 21983, Korea; ezone23@yonsei.ac.kr

**Keywords:** polymeric microsphere, wastewater, vinylic polymer, natural polymer, adsorption

## Abstract

This review addresses polymer microspheres used as adsorbent for wastewater treatment. The removal of various pollutants (including dyes, heavy metal ions, and organic pollutants) is a prominent issue, as they can cause severe health problems. Porous microspheres can provide large specific area and active sites for adsorption or photo degradation. Enhancement in performance is achieved by various modifications, such as the introduction of nanoparticles, magnetic particles, and ZIF-8. Some microspheres were synthesized from synthetic polymers such as vinylic polymer and polydopamine (PDA) through a facile fabrication process. Natural polymers (such as cellulose, alginate, and chitosan) that are biodegradable and eco-friendly are also used. The adsorbents used in industrial application require high adsorption capacity, thermal stability, and recyclability. Batch adsorption experiments were conducted to investigate the optimal conditions, influence of related factors, and adsorption capacities. Insights regarding the adsorption mechanisms were given from the kinetic model, isotherm model, and various characterization methods. The recyclability is investigated through regeneration ratio, or their maintenance of their capability through repeated adsorption-desorption cycles. The high potential of polymer microsphere for the removal of pollutants from wastewater is shown through the high adsorption capacities, environmentally friendliness, and high stability.

## 1. Introduction

Water pollution caused by heavy metal ions and dyes is causing serious environmental problems. Heavy metal ions and dyes such as chromium ion (Cr(VI)) and methylene blue (MB) used in various industries are soluble in aquatic conditions [1,2]. In an aquatic environment, they can be easily consumed and can cause severe health problems, including carcinogenicity and mutagenicity. Various methods have been used for the detoxification and removal of heavy metal ions and dyes, including extraction, flocculant, electrocatalytic oxidation, and membrane separation [3]. Among them, adsorption is an efficient method due to its gentle operating condition, cost-effectiveness, and the fact that it does not derive secondary pollution [4]. Currently, powder, films, and lamellar forms are used, but they have low removal efficiency due to insufficient active sites and low surface area. Activate carbon (AC) is used widely for commercial purpose, but it has drawbacks in that its removal efficiency toward hydrophobic dyes, colloidal dyes, and some macromolecules are low, and it is expensive. Thus, hollow microspheres became promising adsorbents for their high surface area and is convenient to recover after usage [5]. Polymer microspheres can not only enhance the stability and mechanical property of the adsorbents by providing network or protection for other materials but can also enhance the adsorption capacity. To enhance the adsorption capacity, various modifications are put into place. Polymers can provide more active sites and functional groups used for the adsorption. Nanomaterials such as TiO_2_ are used as photocatalyst as they can operate in mild conditions, and they use renewable energy sources [6,7,8]. Various polymers are used to prepare adsorbents to overcome the conventional adsorbents by enhancing the adsorption capacity, recyclability, non-toxicity, cost-effectiveness, and unique application. Thus, eco-friendly, efficient, and cost-effective polymeric microspheres presents their potential as adsorbents for industrial wastewater treatment.

Vinyl polymer-based magnetic nanocomposites are used as catalysts for the removal of dyes and heavy metal ions from aqueous solution. Inorganic/organic nanocomposites can be incorporated into a polymer matrix such as polystyrene (PS), which is easy to handle, economical, and of uniform size [9]. Current studies display low adsorption efficiency for a low initial concentration. Thus, research is done to detect in surface waters at ng/L and µg/L [10]. Poly(glycidyl methacrylate) (PGMA) is used due to its high mechanical strength, pH resistance, and the high reactivity of the epoxy group with adsorbate [11]. Magnetic adsorbents are used to enhance the removal of dyes. They have zero energy separation, do not cause secondary pollution, and have high adsorption efficiency, fast separation speed, and good recyclability. These magnetic nanoparticles present on the surface of porous vinyl polymer microsphere can easily bind with the adsorbate. Coating with diazo resin (DR) can even protect Fe_3_O_4_ in various pH environment and can enhance the adsorption capacity [12].

PDA has been used as microsphere or coating for microsphere for adsorption of organic dyes and heavy metal ions. Dopamine (DA) can spontaneously self-polymerize under weak alkaline environment at room temperature [13]. PDA has excellent adsorbing capacity, and has abundant functional groups like hydroxyl and amine that can bind both organic and inorganic pollutants though various types of interactions. However, adsorbents made of pure PDA display low adsorption capacity [14,15]. These properties can be improved by various types of modifications. The biomimetic, hydrophilic, eco-friendly PDA inspired from mussels can be coated on various substrate and nanoparticles. Additionally, PDA can be synthesized into diverse sizes by controlling the pH of the environment [16,17].

Cellulose is economic, non-toxic, and biodegradable natural, renewable polymer which makes it a promising substance for the preparation of bio-based adsorbent. Cellulose beads can be modified into cellulose-based adsorbents. The separation for batch operation is facile, as it can be handled easily in large volume [18]. Carboxylated cellulose nanocrystals (cCNC) can be used to make cellulose microbeads by spray drying from aqueous suspension. The agglomeration of cCNC that are rescaled to micrometer-size particles, which can be used as adsorbents. The cellulose microbeads can overcome some limitations of activated carbon (AC) (which are more expensive) and yield great amount of CO_2_ during the wastewater treatment process. The spray drying method is also scalable into industrial processes [19].

Alginate is used widely as adsorbents. Sodium alginate (SA) is an eco-friendly, non-toxic, biocompatible, biodegradable natural polymer. It has abundant functionalities such as carboxyl and hydroxyl groups which can be used beneficially for the removal of heavy metal ions and dyes. One of the limitations of SA is its poor mechanical strength, which is very challenging to improve. The interaction of graphene oxide (GO) and SA is one way to solve the issue [20]. SA can also from hydrogel beads to improve the stability [21]. Introducing other metal ions such as Cu(II) can enhance the adsorption efficiency by increasing the adsorption sites [22]. Chitosan is also a biodegradable and non-toxic abundantly available biopolymer which can be used to adsorb various pollutants, especially the anionic contaminants. A microporous chitin microsphere was made by surfactant micelle swelling method and was used for removal of heavy metal ions [23].

This review addresses polymer-based spheres for wastewater treatment, broadly classified into microspheres prepared from (i) synthetic and (ii) natural polymers, and (iii) polymeric spheres used for photo-Fenton process. The proposed mechanism, influencing factors, stability, recyclability, and efficiency are reviewed to understand the enhanced adsorption removal of dyes, heavy metal ions, and organic pollutants. Among polymeric sphere adsorbents, the photocatalysts are particularly divided for their unique features, and photocatalysts prepared from natural polymers are mainly discussed.

## 2. Synthetic Polymer

**Figure 1:**
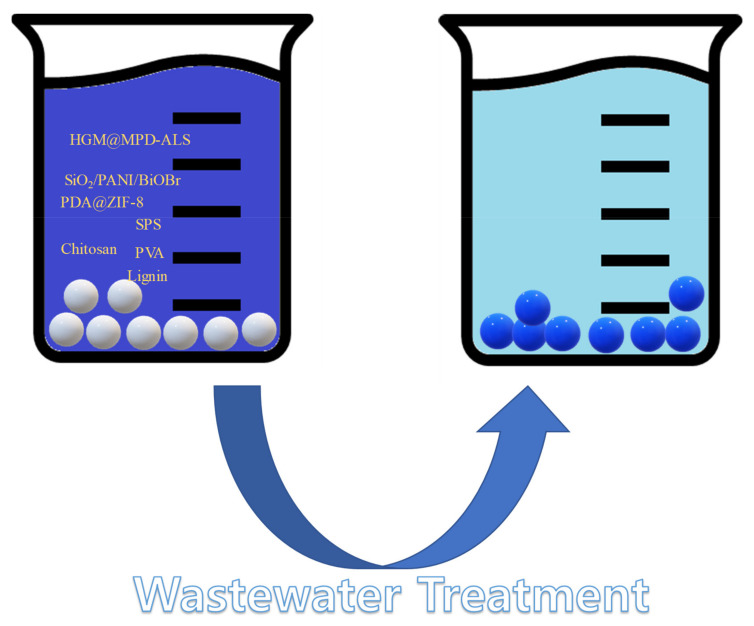
Schematic representation of wastewater treatment.

As Figure 1 represents, microsphere adsorbents derived from polymers are fabricated to purify wastewater. Both synthetic and natural polymers are used for the preparation of microsphere adsorbent. Various synthetic polymers were used, such as graphene oxide, sodium silicate, polymeric resin. Summaries of the efficiencies for each adsorbent are listed in Table 1.

A porous inorganic polymer microsphere was fabricated via suspension dispersion solidification method for removal of Pb^2+^ or other heavy metal ions from wastewater [24]. SEM analysis confirmed that the microsphere had a diameter of around 100 μm. It was added with a foaming agent for formation of pore structures, which enhanced the BET surface area from 79.80 m^2^/g to 100.9 m^2^/g and decreased the pore diameter to 7 nm. Due to its small particle size and large surface area, the microsphere had a high adsorption capacity of 629.21 mg/g for Pb^2+^. In addition, adsorption reached equilibrium within 5 h due to the high adsorption rate and low diffusion resistance. During the adsorption, the adsorbates moved through the pores of the microsphere to the center, so all the active sites were used. The adsorption process followed the pseudo-second-order (PSO) model, indicating the chemical adsorption. It followed the Langmuir model and the D-R adsorption model, suggesting the monolayer’s adsorption, which was due to a combination of chemical and physical adsorption. It could be easily regenerated by using EDTA-2Na solution with 51% of regeneration ratio.

A multi-functional HGM@MPD-ALS adsorbent with inorganic core and surface modified with organic functional groups was fabricated to treat dyeing wastewaters [25]. The adsorbent was synthesized by co-grafting m-phenylenediamine (MPD) and sodium allyl sulfonate (ALS) on the surface of a hollow glass microsphere (HGM). Alkali corrosion increased the surface roughness and –Si–OH groups of HGM, and functional groups were grafted onto the surface of HGM. The obtained microsphere had amphoteric attraction abilities for anionic and cationic dyes, acid green 25 (AG25), and fuchsin (BF) dyes, respectively. The adsorption followed the PSO kinetic model and the Langmuir isotherm model. The maximum adsorption capacity for AG25 was 454.55 mg/g and 588.24 mg/g for BF. XPS peaks were analyzed to examine the adsorption mechanisms (which were electrostatic attraction, π-π stacking, and hydrogen bonds), which were assumed to be the interaction forces of adsorption. From the hollow structure of HGM, the material has self-floating ability; thus, adsorbents over 95.7% were adsorbed at water surface through a self-floating process within 12 min. This was very helpful for the solid–liquid separation process and recycling of adsorbent, which are very important adsorbent properties for a longer lifetime. Another organic-inorganic hybrid of β-cyclodextrin-graphene oxide aerogel microspheres (CD-GAM) was fabricated to remove dyes and organic micropollutants from water [26].

**Figure 2:**
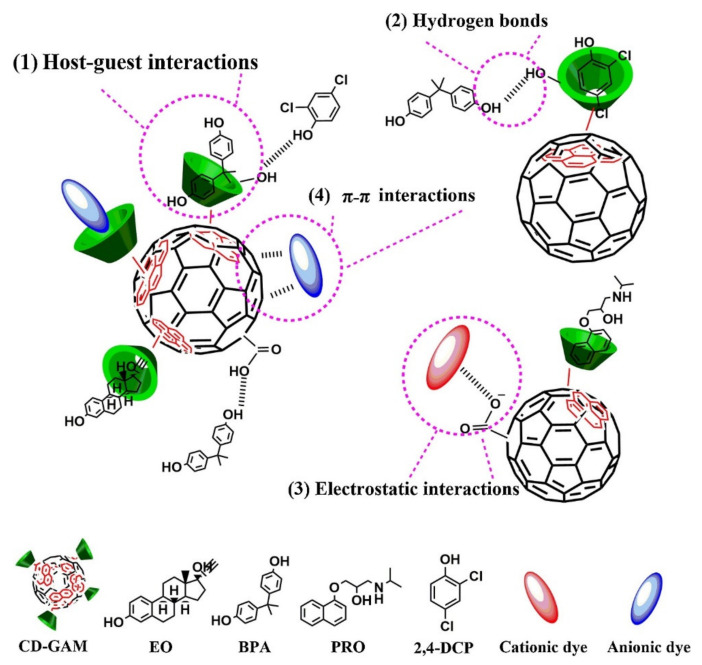
Schematic illustrating the adsorption mechanisms of CD-GAM for dyes and organic micropollutant (Reproduced with permission from Nie et al. [26], Copyright 2021, Elsevier Ltd.).

Figure 2 represents the schematic representation of removal of pollutants. CD-GAM was prepared via the electrospraying method combined with freeze-casting and successive mild self-assembly. The TGA curve of CD-GAM showed gradual slope of weight loss below 100 °C due to loss of water but had rapid loss near 300 °C due to CD loss. Adsorption for various adsorbates was possible due to graphene oxide and β-cyclodextrin that were integrated into aerogel microsphere. The adsorption capacities for dyes were 439 mg/g for MB, 388 mg/g for rhodamine B (RhB), 234 mg/g for acid red 87, and 167 mg/g for methyl orange (MO). Additionally, it showed great adsorption capacities for organic micropollutants aromatic model compounds and pharmaceuticals; they were 17 mg/g for carcinogenic 2,4-dichlorophenol, 49 mg/g for propranolol hydrochloride, 19 mg/g for contraceptive ethynyl estradiol, and 38 mg/g for plastic components bisphenol A. Most pollutants reached equilibrium within 10 min, and the CD-GAM microsphere maintained its adsorption capacity over 80% during five cycles. In order to check the role of carbon material, a porous coal gangue microsphere/geopolymer (CG/KGP) composite was made for filtrating wastewater and adsorption treatment of dye [27]. It was synthesized by adding coal gangue microspheres into geopolymer matrix. CG/KGP composites mainly contain amorphous phases, and functional groups are mainly on the particle surface. XRD patterns confirmed that when the CG/KGP composite was pre-calcined below 1000 °C, the CG microspheres are consisted of kaolinite and α-quartz phases, homogeneously dispersed, and bonded well with pure KGP. Incorporation of CG microspheres, especially CG800 and CG900, greatly enhanced the adsorption capacity and BET surface area. The CG900/KGP samples showed 24.6 mg/g adsorption capacity and the removal efficiency achieved 98% at the dosage of 4 g/L. The adsorption of MB followed the PSO kinetic model and the Langmuir model. The maximum adsorption capacity of CG/KGP particles reached around 10 mg/g. A polymer coated magnetic nanoparticle, PMMA@Fe_3_O_4_@DR composite, was prepared for simple and quick dye adsorption from wastewater [28]. First, a PMMA (polymethylmethacrylate) porous microsphere was synthesized via the seed swelling method. Then, iron oxide nanoparticles were formed in situ within the pore of the microsphere. Finally, the porous magnetic microspheres were encapsulated with photosensitive diazo-resin (DR) to increase the stability of the magnetic material. The magnetic measurements were measured using an alternate gradient magnetometer. The magnetization was 4.85 emu/g in magnetic field of −4.5 to 4.5 kOe at room temperature. TGA curves revealed that the main decomposition occurred at 250–400 °C. Different properties of the magnetic microsphere proved that the microsphere was a reusable adsorbent for the efficient removal of anionic and aromatic dyes by binding the anionic organic compounds to porous magnetic microsphere during wastewater treatment. Its magnetic and adsorption properties were also maintained, even in harsh conditions. Another magnetic microsphere as adsorbent for cationic dyes is made from polyacrylamide (PAM) [29]. The porous magnetic PAM microsphere was prepared using the phase separation method induced by polymerization. The adsorption capacity of methylene blue increased from 263 mg/L to 1977 mg/L as initial concentration increased from 5 mg/L to 300 mg/L. The adsorption capacity increased with increasing initial pH and reached a maximum of 1990 mg/g at pH 8, indicating the electrostatic interaction between the microsphere and dye. The microsphere also showed high adsorption capacities for other dyes (1937 mg/g for neutral red (NR) and 1850 mg/g for gentian violet) and the mixed-dye solution. Complete adsorption was possible even for a very low initial concentration by using a different pore structure PAM microsphere with different PEG concentrations. The TGA curve showed that 70 wt% weight loss occurred at 200–500 °C. The adsorption followed the Langmuir isotherm model. The dye-absorbed microsphere could be easily desorbed and used for six cycles without resulting in a loss of adsorption capacity. Mesoporous microsphere made up of melamine formaldehyde resin (MMFRS) without any inorganic materials is also made. MMFRS was fabricated for the removal of organic compound perfluorooctanoic acid from wastewater [30]. It was fabricated through suspension polymerization. Its mesoporous property with an average pore size of around 10 nm (analyzed from the TEM image) and its large anion-exchange capacity of 0.3 mmol/g make it a promising adsorbent of perfluoroalkyl and polyfluoroalkyl substances, particularly perfluorooctanoic acid from wastewater. TGA curves confirmed that the greatest mass loss occurred at temperatures between 400 and 800 °C. The sorption process followed the PSO kinetic model, and the sorption isotherms followed the Freundlich and Langmuir model. The absorption amount of MMFRS exceeded the current commercial powdered activated carbon, as it reached equilibrium within 24 h and could be used in broad pH range. MMFRS is also easily and economically regenerated by using a 7.5 mM dilute of NH_3_·H_2_O, and it reached over 85% of regeneration percentage after 20 manifold cycles.

**Figure 3:**
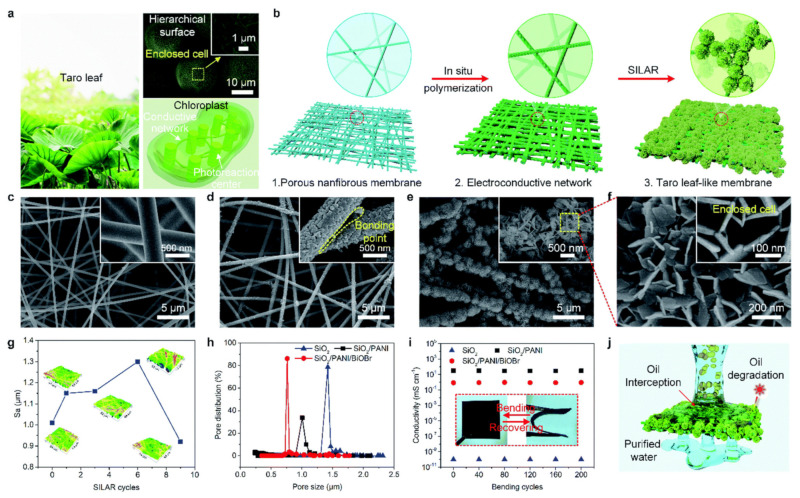
(**a**) Photographs showing the taro leaf with superwettability due to a hierarchical surface and photosynthesis through a chloroplast. (**b**) Schematic illustrating the preparation of the taro leaf-like nanofibrous membrane. SEM images of the (**c**) SiO_2_, (**d**) SiO_2_/PANI, and (**e**) SiO_2_/PANI/BiOBr nanofibrous membranes. (**f**) Ultrathin BiOBr nanosheets coated on the SiO_2_/PANI/BiOBr nanofiber surface, which form numerous enclosed cells. (**g**) 3D height map and Sa of SiO_2_/PANI/BiOBr nanofibrous membranes with different numbers of SILAR cycles. (**h**) Pore size distribution and (**i**) conductivity of different nanofibrous membranes. (**j**) Schematic illustrating emulsion separation and self-cleaning capacity of the SiO_2_/PANI/BiOBr membrane (Reproduced with permission from Zhang et al. [32], Copyright 2021, Royal Society of Chemistry).

The degradation of pollutants is another way to get rid of pollutants, and TiO_2_ is an excellent semiconductor used for photocatalysis. It was incorporated into a poly[ANE + N-PMI] microsphere to decompose carcinogenic compounds into harmless product [31]. The biomass polymer microsphere was prepared via precipitation polymerization. Amino-modified TiO_2_ nanoparticles were covalently linked to the surface of the biomass-driven phenylpropenes trans-anethole (ANE) and N-phenylmaleimide (N-PMI) polymer. The microspheres were used as photocatalysts for the degradation of RhB dyes and tetracycline (TC) antibiotics. The microsphere and the TiO_2_ nanoparticles were covalently bonded, and the microspheres participated in the electron transmission at the surface of the nanoparticles to minimize the recombination between electrons and holes. The intensity of the photoluminiscent (PL) spectroscopy peak at 480 nm (which was lower than that of single TiO_2_) indicates the low recombination rate. The large surface area of microsphere provided more active sites for degradation. Superoxide free radicals (·O_2_^−^) and hydroxyl radicals (OH) were the primary and secondary reactive species during photo-degradation. The degradation rate of microsphere reached 95% for 50 mg/L RhB and 97% for 100 mg/L TC. The degradation process followed pseudo-first-order (PFO) kinetics. In order to check the effectiveness of photocatalysis, membranes with photoactive microspheres were linked to conducting composite nanofiber. Inspired by multiscale structures of taro leaf, as shown in Figure 3, a biomimetic nanofibrous membrane composed of BiOBr microspheres anchored on electroconductive SiO_2_/PANI core-shell nanofiber was fabricated to solve issues of prevailing oil separation membranes that suffer oil fouling issues [32].

The membrane shows great superhydrophilicity, underwater superoleophobicity, and low oil adhesion due to the surface multiscale structures and hydrophilic matrix. The matrix displays high permeation flux of 6140 L/m^2^ h, and high separation efficiency at total organic carbon content below 5 mg/L, which can be ascribed to the properties of biomimetic structure (which are the submicron pore size, high porosity, and good channel interconnectivity). The low PL spectra intensity indicates a low recombination rate of electrons and holes. The presence of BiOBr/PANI heterojunction arrays with visible light catalytic activity and 3D PANI electroconductive networks that serve as hole conducting pathways facilitate self-cleaning properties, retaining a high flux recovery ratio over 98%.

### 2.1. Vinylic Polymer

Adsorbents derived from vinylic polymer such as divinylbenzene (DVB) or styrene-divinylbenzene (St-DVB)-based microsphere are often used. St-DVB have high porosity and chemical and mechanical stability. They usually show high adsorption capacities toward heavy metal ions, as shown in Table 2. *Bacillus velezensis* was immobilized on the surface of polyvinyl alcohol (PVA) microsphere to remove organics in slaughter wastewater. *Bacillus velezensis* was isolated and genetically identified as carbon oxygen demand (COD)-degrading bacteria [33]. FT-IR analysis confirms the crosslinked network structures of PVA microsphere, and SEM imaging showed the surface structure of carrier microsphere. The removal rate of CODMn was 16.99% within 24 h at 37 °C, pH 7.0, and 120 rpm, which was 2.2 times that of free bacteria. The COD degradation rate was higher than that of the PVA microsphere without immobilization (20.08 to 10.81, respectively), and the processing time reached 36 h. The digestibility of starch was 20.1% and the protein digestibility was 17.5%, which was higher than that of free bacteria, which was 42.2% and 37.2%, respectively. The obtained microsphere showed enhanced organic removal in the slaughter wastewater from its synergic effect of adsorption and biodegradation.

Poly(ethylene glycol dimethacrylate-n-vinyl imidazole) (poly(EGMDA-VIM)) microspheres were fabricated for the removal of phenol from an aqueous solution [34]. The microsphere was synthesized via suspension polymerization and characterized by various analysis techniques. The microsphere had high specific surface area of 304.4 m^2^/g. The adsorption followed the PSO kinetic model and the Freundlich isotherm model, and it was an exothermic and spontaneous process. The maximum adsorption capacity for phenol was 34.7441 mg/g at 25 °C and neutral pH from the Langmuir model. Poly(EGMDA-VIM) microsphere was easily regenerated by using 0.01 M NaOH solution and could be reused for five cycles with maintained adsorption capacity. To improve adsorption properties, carbon can be incorporated into the polymer matrix. Phenolic resin-based microfiltration carbon membranes (MFCMs) were prepared by incorporating polyacrylonitrile-based microspheres to effectively separate the emulsion of oil and water [35]. The TGA result confirmed that incorporating polyacrylonitrile (PAN) improved the thermal degradation property of precursor, and that the PAN amount should exceed 25%. With increased PAN, the average macropores of resultant MFCMs decrease, mesopores and micropores were reduced, and the microstructure was densified. The pore size distributions were measured via nitrogen adsorption analyzer. PAN microsphere incorporation improved emulsified oil–water separated performance and was stable for over 240 min. The oil–water separation is dependent on early-stage adsorption and size exclusion at steady state. The obtained MFCMs containing 35% PAN microsphere achieved 93.6% oil removal, along with the permeation flux of 251.1 kg/m^2^h under the feed oil concentration of 50 mg/L, trans-membrane pressure at 0.03 Mpa, and feed flow rate at 6 mL/min. As feed oil concentration or trans-membrane pressure increased, the permeation flux increased.

Divinylbenzene (DVB) and polystyrene (PS)-based polymers are often used as adsorbents for heavy metal ions and dyes. A porous (Styrene-divinylbenzene)/CuNi bimetallic nanocomposite microsphere (P(St-DVB)/CuNi BNC) was fabricated as an adsorbent for heavy metals in drinking water treatment [36]. It was prepared via oil-in-water emulsion polymerization of styrene and divinylbenzene while CuNi bimetallic NPs existed. SEM image showed that the average diameter of the nanocomposite is about 112 μm. TEM imaging showed that the nanocomposite is entrapped in a spherical shape. The adsorption efficiencies of Pb^2+^, Cd^2+^, Mn^2+^, and Zn^2+^ ions from synthetic water were evaluated via batch study. The removal efficiency increased as contact time and adsorbent mass increased but decreased as initial metal concentration increased. Adsorption differed for each metal, and P(St-DVB)/CuNi BNC showed the highest adsorption efficiency for Mn^2+^ and Zn^2+^. It also differed regarding the pH range. While lead showed the best adsorption at pH 5–7, Cd, Mn, and Zn were optimal at pH 7. The maximum adsorption capacity for each metal was 15.60 mg for Pb(II)/g, 5.28 mg for Cd(II)/g, 22.42 mg for Mn(II)/g, and 20.57 mg for Zn(II)/g, respectively. The adsorption process followed the PFO kinetic model and the Langmuir adsorption isotherm model. Another microsphere using St-DVB was modified by introducing attractive thiol (SH) groups on the surface of polymeric matrices for the removal of heavy metal ions in wastewater [37]. The obtained St-DBV-SH microsphere was fabricated using a four-step modification. First, parent St-DVB microspheres were treated by H_2_SO_4_ to obtain thiol derivatives. The oleum was then added, and sulfonic groups were converted to chloride acylsulfonic groups while PCl_5_ and POCl_3_ existed. Lastly, the microsphere was reduced by using SnCl_2_·2H_2_O. The modification allowed a thiol group with 12.94% of sulfur on the surface of microsphere, and it had low swelling tendency in organic solvents of 8% (at maximum). The presence of thiol groups was confirmed via elemental analysis. DSC analysis showed lowered thermal resistance after modification, but it was sufficient for sorption process. The batch method was conducted to evaluate the removal of Cu(II), Zn(II), Cd(II), Pb(II), and Ni(II). For all adsorption studies, pH 5 was the optimal condition. The adsorption followed the PSO kinetic model and the Langmuir isotherm model, which was confirmed by high values of determination coefficients. Similarly, a monodisperse sulphonated polystyrene (SPS) microsphere was prepared to check the effect of the sulfonic group for the sustainable elimination of heavy metals in wastewater [38]. It also followed the PSO kinetic model and the Langmuir isotherm model. The SPS microsphere was fabricated using a scalable method by sulphonated modification, having a considerable number of sulphonic groups on the surface of the microsphere (which provided efficient binding sites for metal ions). The abundant sulphonic acid groups and their superior monodisperse properties increased the adsorption ability in both quantity and speed. The theoretical maximum capacities at pH 3.5 and 30 °C were 49.16 mg/g for Pb^2+^, 15.38 mg/g for Zn^2+^, and 13.89 mg/g for Cu^2+^, respectively. The adsorption equilibrium for Pb^2+^ was reached within 1 min. The peak of sulfuric acid groups from XPS analysis showed that the strong interaction between sulfuric acid groups and metal ions is the main cause of the great adsorption capacity. The micron structure of SPS microsphere resolved the excessive hydrophilia issue caused by rich sulphonic acid groups and easily separated; thus, the microsphere has unparalleled advantages regarding nanoscale adsorbents. The microsphere maintained good recycle capacity after five regeneration cycles without any loss in adsorption capacity.

Organic–inorganic materials with spherical shapes consisting of DVB and triethoxyvinylsilane (TEVS) were synthesized and investigated for the removal of organic compounds from wastewater [39].

**Figure 4:**
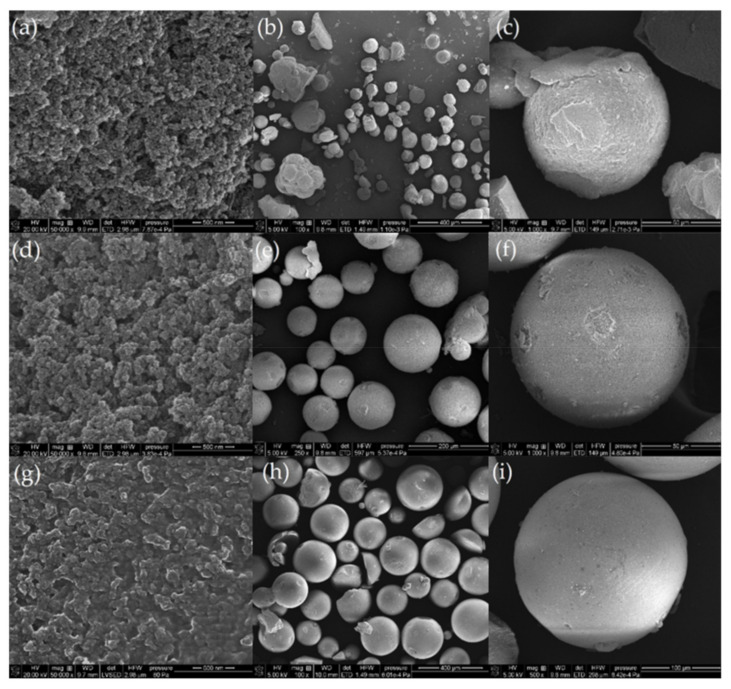
SEM images of DVB:TEVS = 1:2 (**a**–**c**), DVB:TEVS = 1:1 microspheres (**d**–**f**) and DVB:TEVS = 2:1 microspheres (**g**–**i**) in different magnification (Reproduced from Bosacka et al. [39], 2021, MDPI).

DVB was used as a crosslinking monomer and TEVS was used as a silane coupling agent, and the influence of their molar ratio was investigated. As shown in Figure 4, SEM image shows that the DVB:TEVS = 1:2 ratio microsphere was largely deviated from the ideal spherical form, and a 2:1 microsphere ratio showed a regular shape with a uniform and smooth surface. The adsorption properties were also different toward nitrobenzene, 4-nitrophenol, and phenol. For all microspheres, nitrobenzene showed the strongest decrease of adsorbate concentration and the lowest decrease for phenol, which is related to adsorbate solubility/hydrophobicity. For nitrobenzene, 2:1 microsphere showed the greatest adsorbate loss, while for 4-nitrophenol and phenol, 1:1 microsphere showed the greatest loss. TG and DTG curves showed that DVB-TEVS materials are thermally stable up to 330 °C and that the main destruction occurs over 450 °C, indicating their applicability in a comparatively wide temperature range. The remarkable selectivity toward various organic adsorbates makes it a promising adsorbent. Finally, the effect of ionic liquid in the polymeric microsphere was tested in regards to wastewater treatment. Pyridine polyionic liquid porous microspheres (PPLM) were prepared for the selective adsorption of anionic organic dyes from industrial wastewater [40]. A P(VP-DVB)-6 microsphere was chosen as the optimal adsorbent based on adsorption conditions and operational requirements ascribed to its optimal comprehensive properties. The adsorption capacities for Congo Red (CR), Bromophenol blue (BPB), Sunset Yellow (SY), Alizarin red R (AR), Safranine T (ST), and MB of P(VP-DVB)-6 microsphere were evaluated via UV-vis spectrophotometer, and it was found that it had outstanding selective adsorption effects for three anionic dyes (CR, SY, and BPB). The absorptions for SY and BPB were further investigated and fitted to the PSO kinetic model. The adsorption rate was affected by the concentration of both dye and adsorbent. The whole adsorption process occurred through two states: the surface adsorption and the intraparticle diffusion. The adsorption fit the Langmuir isotherm model, indicating that the adsorption was single molecular layer adsorption.

### 2.2. Polydopamine

Polydopamine (PDA) is often chosen as adsorbents due to its abundant functional groups and the fact that it can easily coat various substances. PDA-based microspheres mostly showed high adsorption capacities toward MB dye and Cr(VI) ions, as shown in Table 3.

H-PDA-MCs (Hollow polydopamine microcapsules) were synthesized via the oxidation self-polymerization of dopamine, using a Fe_2_O_3_ nano-cube as a template, to purify organic wastewater by capturing organic dyes [41].

**Figure 5:**
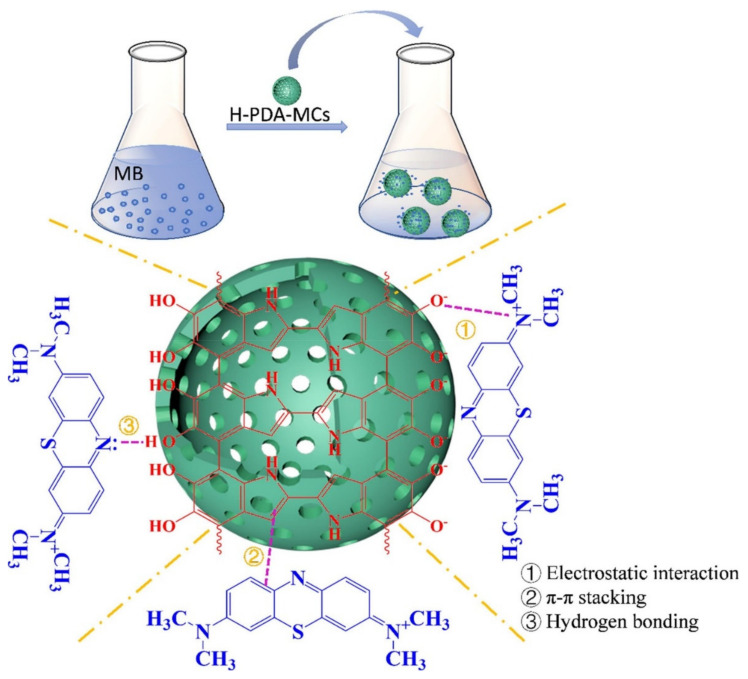
Schematic illustration of the possible interaction mechanism between the H-PDA-MCs and MB (Reproduced with permission from Feng et al. [41], Copyright 2021, Elsevier Ltd.).

The obtained microcapsule had a hollow, mesoporous structure with a large specific surface area of 24.4 m^2^/g and it had an electronegative surface under neutral or alkaline conditions. The results of a batch adsorption experiment show that it had an adsorption capacity for MB of 191.55 mg/g with a short equilibrium time of 60 min at 25 °C. The adsorption process follows the Temkin and Langmuir isothermal linear models and was a spontaneous and endothermic process. The effective capture of MB results from multiple interactions of electrostatic interaction, hydrogen bonding, and π-π stack as shown in Figure 5. To investigate the adsorption mechanism, UV-vis spectra was used. The adsorption peak of MB in the microsphere showed a bathochromic shift compared to the pristine MB due to the interaction between the microsphere and the dye. PDA microspheres prepared using a similar oxidative polymerization method was tested for adsorption of MB from aqueous solution [42]. TEM imaging showed that the microsphere had an average diameter of around 600 nm, which results in a large specific surface area. MB adsorption was highly dependent on the initial solution pH, temperature, initial MB concentration, and contact time. The adsorption capacity of the obtained microsphere was 90.7 mg/g at 25 °C, and its high performance is driven from the π-π stacking, electrostatic interactions, and the structure properties of PDA microspheres (which is supported by the FTIR results). Its monolayer adsorption capacity was 88.89 mg/g. Additionally, the adsorption process took two steps, and the intraparticle diffusion was not the rate-limiting step. Thermodynamic analyses revealed that the adsorption was spontaneous, endothermic, and a physisorption process. Another PDA microsphere was fabricated for the selective adsorption and separation of organic dyes [43]. The PDA microsphere was synthesized via oxidation polymerization under basic conditions. Its adsorption capacity for nine water-soluble dyes was evaluated. It showed high, efficient adsorption ability toward cationic dyes (MB): malachite green (MG) and neutral dye (NR). However, it showed low ability towards cationic dyes (safranine T (ST), and RhB), and almost no adsorption ability for anionic dyes: eosin-Y (EY), eosin-B (EB), acid chrome blue K (ACBK), and MO. UV-vis spectroscopic spectra revealed that the chemical structures of dye molecules are the cause of varying adsorption abilities for different dyes. The adsorption for cationic and neutral dyes followed the PSO model and the Langmuir isotherm model. The selective adsorption of the PDA microsphere has potential in separating mixture of dyes in aqueous solution. The above three PDA microspheres for cationic dye adsorption all followed the PSO model and the Langmuir isotherm model, indicating the monolayer adsorption. In addition, electrostatic interaction and π-π interaction were the main mechanisms of the adsorption. PDA based microspheres are also used as adsorbents for metal ions. A PAM/PA/PDA hydrogel-based electrochemical sensor for the detection of Cu^2+^ ions was prepared through interactions between the DA, acrylamide (AAM), and phytic acid (PA) monomers [44]. They were made into microstructured 3D network to increase the surface area for an enhanced number of immobilized molecules and ions, as well as high conductivity. EIS was used to investigate the interfacial resistances. The PAM/PA/PDA hydrogel had a very low resistance of 0.3 kΩ, proving its superb electrical conductivity. The PAM/PA/PDA sensor showed a low detection limit of 1 nmol/L and a wide linear range. The hydrogel also showed a high adsorption capacity towards Cu^2+^ ions (231.36 ± 4.70 mg/g). The adsorption mechanism was inferred using XPS, which indicated that Cu^2+^ ions strongly interact with hydroxyl and carboxyl group of the obtained hydrogel. The sensor made from PAM/PA/PDA hydrogel shows a high selectivity toward Cu^2+^ ions and high reproducibility. Therefore, it can be used for both the detection and removal of Cu^2+^ ions from aqueous condition. Besides incorporating other substances, the adsorption capacity of the PDA microsphere can also be enhanced by controlling the size of microsphere. A PDA microsphere inspired by mussels was prepared into controllable sizes through in situ oxidative polymerization at air condition by adjusting alkaline solutions (such as ammonia [45]). With lower amounts of ammonia, the PDA microsphere is likely to have a larger size. Dynamic light scattering (DLS) analysis was measured for investigation of particle size distribution, which were mostly in the range of 520–570 nm. The adsorption of Cr(VI) reached equilibrium within 8 min, at an optimal pH range of 2.5 to 3.8. The PDA microsphere showed remarkable sorption selectivity while competing ions of SO_4_^2–^, supported by XPS results, NO_3_^–^ and Cl^–^ coexisted at high extent, due to the formation of the strong amine and carbonyl/hydroxyl group complexation. Due to well-dispersed morphology and a strong affinity between Cr(VI) and catechol or amine groups, the adsorption capacity reached 42,000 kg/kg sorbent, and wastewater could be reduced from 2000 ppb to below 50 ppb, meeting the drinking water criterion. One kg sorbent can purify around 100 t Cr(VI) contaminated wastewater, and efficiently regenerated by binary alkaline and salts mixtures. A core-double-shell structured magnetic polydopamine@zeolitic imidazolate framework-8 (MP@ZIF-8) hybrid microsphere was also prepared for the synergistic reduction and adsorptive removal of Cr(VI) [46]. The morphology and structure were confirmed using SEM/TEM images. The MP@ZIF-8 microsphere has a core of magnetic Fe_3_O_4_ nanoparticles, a PDA layer as an inner shell, and a porous ZIF-8 nanocrystal as an outer shell. Magnetization hysteresis loops were also characterized, and the results showed that the microsphere had a low magnetization saturation value of 59.4 emu/g. It showed an improved Cr(VI) removal capacity of 136.64 mg/g, higher than that of pristine magnetic PDA, which had a Cr(VI) removal capacity of 92.27 mg/g. The removal kinetics followed the PSO kinetic model and were heavily dependent on pH values. During the adsorption process, XPS analysis revealed that the reduction of nitrogen atom group on ZIF-8 and PDA facilitated the conversion of Cr(VI) into the much less toxic Cr(III), and then the Cr(III) was immobilized on the MP@ZIF-8. The size-controllable PDA microsphere showed a higher adsorption capacity than a magnetic PDA microsphere with ZIF-8.

## 3. Natural Polymer

Natural polymers such as lignin, cellulose, or chitosan are often used, or synthetic microspheres are modified using a bio-based polymer such as vanillin. Natural polymers meet one of the requirements to minimize the secondary pollution in that they are mostly eco-friendly and biodegradable. Besides this, natural polymers also exhibit great performance as adsorbents (which is shown in Table 4).

**Figure 6:**
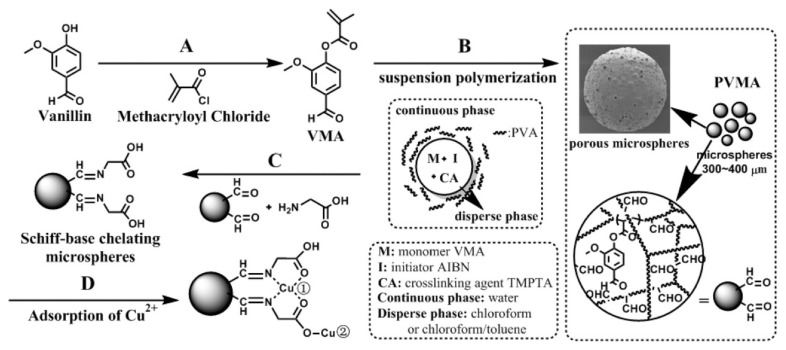
Schematic strategy for preparing polymeric microspheres starting from vanillin (**A**,**B**), for preparing Schiff-base chelating microspheres by reaction with glycine (**C**) and for performing adsorption by the microspheres toward Cu^2+^ ions (**D**) (Reproduced with permission from Zhang et al. [48], Copyright 2016, American Chemical Society).

Natural polymers require extraction from their natural state. Thus, a new technique to directly extract lignin microsphere from highly alkaline black liquor was developed by lowering the pH under 4 and hydrothermal treatment [47]. Native lignin has the tendency to aggregate in solvents and has low adsorption capacities for metal ion or drugs. However, nano/microstructured lignin shows a much better performance due to increased surface area. Due to the strong adhesive property of lignin to cellulose, delignification and purification processes were required to isolate lignin. Field-Emission SEM (FESEM) imaging showed that the obtained microsphere had uniform poly-disperse morphology, a smooth surface, a relatively uniform size, the typical thermal stability of lignin, and great carbon content around 50–60%. The method resolved the problems of extraction; it was upscalable and was low cost, the extraction efficiency was 15.87 to 21.62 g/L, and the average size of microspheres was around 1 μm. The DTG profile revealed that highest weight loss rate for cellulose happened at 330 °C. The direct extraction of lignin microspheres from black liquor in large scale can be applied as agricultural drug delivery agents, heavy metal adsorbent, and UV absorbers for sun block. After extraction, natural polymers can be used as adsorbents. A polymer derived from vanillin methacrylate (VMA) monomer named poly vanillin methacrylate (PVMA) microsphere was fabricated for the adsorption of Cu^2+^ ions. Figure 6 explains the preparation process. The microsphere was fabricated via suspension polymerization of VMA and the monomer provided by methacryloyl chloride reacted with vanillin, a biomass. The polymerization was conducted in aqueous media and had a high yield of a microsphere over 90% [48].

The microsphere was endowed with surface pores by controlling the nitrogen bubbling mode and optimizing the cosolvent for dissolving the solid monomer. The reactive aldehyde group of microspheres reacted with glycine, being a Schiff-base chelating material. The Cu^2+^ concentration was measured via atomic absorption spectrophotometer and the microsphere showed a great adsorption capacity of Cu^2+^ at a maximum of 135 mg/g. The adsorption process was chemical adsorption, and the Schiff-base structures and the carboxylic groups both contributed to adsorption. TGA analysis confirmed that the polymer chains decomposed in the range of 250 to 480 °C.

**Figure 7:**
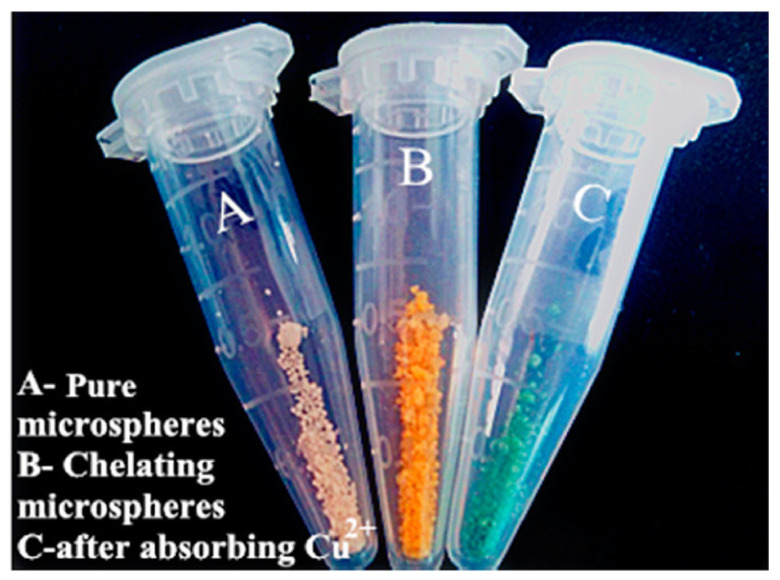
Typical photographs of pure PVMA microspheres, chelating microspheres, and the microspheres after adsorbing Cu^2+^ (Reproduced with permission from Zhang et al. [48], Copyright 2016, American Chemical Society).

Figure 7 shows successful adsorption from the color change of the microsphere before and after adsorption. The microsphere can be used as green adsorbents for the treatment of wastewater and can also be used as biomaterials for enzyme immobilization. The microsphere can also be prepared using other lignin derivatives instead of vanillin, such as syringaldehyde and p-hydroxy benzaldehyde. For the removal of uranium from wastewater, a hydroxyapatite (HAP) microsphere adsorbent was fabricated using a template-free hydrothermal method [49].

**Figure 8:**
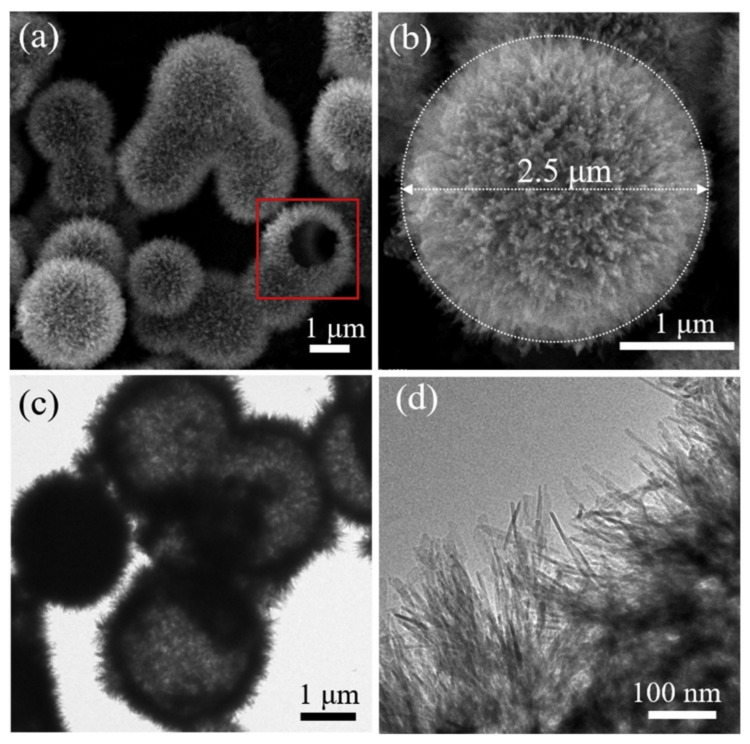
Field emission scanning electron microscope (FE-SEM) (**a**,**b**) and transmission electron microscope (TEM) (**c**,**d**) images of the hollow hydroxyapatite microspheres, showing the hierarchical and hollow structure of the as-prepared hydroxyapatite samples obtained (Reproduced with permission from Wu et al. [49], Copyright 2019, Elsevier Ltd.).

XRD analysis, SEM images, and TEM images as shown in Figure 8 revealed that HAP was crystallized in a hexagonal structure and had a hollow and hierarchical nanostructure. It had a large surface area; the BET surface area was 182.6 m^2^/g and the average pore size was 10.5 nm, indicating efficient removal of U(VI) from wastewater within 5 min ascribed to the abundant active sites that the HAP microspheres provide. The uranium concentration was measured though a uranium analyzer. The XRD peaks showed that the surface chemisorption between U(VI) and HAP formed new uranium-containing compound named autunite (Ca(UO_2_)(PO_4_)_2·_3H_2_O), which is assumed as main sorption mechanism. The adsorption process of both PVMA microsphere and HAP microsphere followed Freundlich isotherm and PSO kinetic model.

Amine terminated sodium alginate/poly-N-isopropyl acrylamide (NH_2_-SA/PNIPA) thermosensitive hydrogel microsphere was fabricated for treatment of heavy metal ions in wastewater [50]. NH_2_-SA/PNIPA showed high, but different adsorption capacity for copper and cadmium ions, due to their different hydration. The concentrations of metal ions were measured via atomic absorption spectrophotometer. The adsorption capacity for Cu(II) was 57.5 mg/g at 20 °C and 56.7 mg/g at 40 °C. The adsorption capacity for Cd(II) was 100.5 mg/g at 20 °C and 88.6 mg/g at 40 °C. Higher temperatures facilitated the adsorption process, but the adsorption capacity of NH_2_-SA/PNIPA decreased at temperatures above the LCST because NH_2_-SA/PNIPA became hydrophobic and its volume shrank. Diffusion coefficient D_s_ was obtained by HSDM (homogeneous surface diffusion model), 4.75 × 10^−11^ m^2^/s at 20 °C and 3.75 × 10^−11^ m^2^/s at 40 °C for Cu(II), and 3.24 × 10^−11^ m^2^/s at 20 °C and 1.84 × 10^−11^ m^2^/s at 40 °C for Cd(II). The adsorption process followed the PSO model and the Langmuir isotherm model, and the process was mainly controlled by complexation reaction. The microsphere could remain its adsorption performance for five cycles. Polyethyleneimine modified magnetic porous cassava residue microspheres (PEI/HMPCR) was prepared for adsorption removal of Cd(II) from wastewater [51]. PEI/HMPCR was prepared via reverse emulsion polymerization with pretreated cassava residue (PCR) as raw material, modified by linear PEI and PEG2000 as porogen. Addition of PEG2000 increased surface area of HMPCR from 8.08 m^2^/g to 17.06 m^2^/g; however, after the addition of PEI, the surface area declined to 14.86 m^2^/g with a pore diameter size of 14.24 nm. The results of FT-IR and XPS analysis indicated that Cd(II) adsorption was mainly with –NH and –NH_2_ groups on the PEI/HMPCR surface, and PEI/HMPCR adsorbent exhibited superparamagnetic with saturation magnetic susceptibility of 9.5 emu/g. TGA results indicate that the second stage of the three weight loss stages at 200 to 450 °C is the main weight loss stage. The maximum adsorption capacity was 143.6 mg/g at pH 6.0, 35 °C and an initial concentration of Cd(II) of 250 mg/L. The introduction of PEI generated more –NH and –NH_2_ groups as adsorption sites for Cd(II). The adsorption process also followed the PSO model and the Langmuir isotherm model. Chemisorption was the determining step, accompanied by a physical process. NH_2_-SA/PNIPA and PEI/HMPCR have similar adsorption mechanisms, but the maximum adsorption capacity for Cd(II) is higher using PEI/HMPCR adsorbent. Additionally, the vinyl polymer microsphere can be stabilized by natural substances. Porous microspheres fabricated from the double emulsion template showed outstanding performance as adsorbent for the removal of cationic dyes [52]. The current method of stabilizing the double emulsion by block copolymer has limited yields. Thus, O_1_/W/O_2_ double emulsion was stabilized with biological surfactant, the particle–pine pollen, through one-step phase inversion. The porous microspherical adsorbent was fabricated via thermal-initiated polymerization of the intermediate phase of O_1_/W/O_2_ double emulsion. SEM imaging showed that the diameter of microspheres was in the range of 50.7 μm to 82.3 μm and had an ellipsoidal shape with an average pore size of 2.6 μm and had an interconnected pore structure. Formation and stabilization of double emission were affected by the volume fraction of oil phase, pollen content and electrolyte concentration. The adsorption capacity of MB was 668.96 mg/g and 749.69 mg/g for methyl violet within 30 min at 25 °C, ascribed to its high dispersibility and the abundant porous structure.

### 3.1. Cellulose

Cellulose is abundant in nature, has strong mechanical strength and abundant surface functional groups (hydroxyl groups), and is easy to modify. It is also biodegradable, being non-toxic (as are most natural polymers). Due to its beneficial properties, it is an adequate candidate for adsorbent of MB. The efficiency of its use as adsorbents and their functional groups is summarized in Table 5.

**Figure 9:**
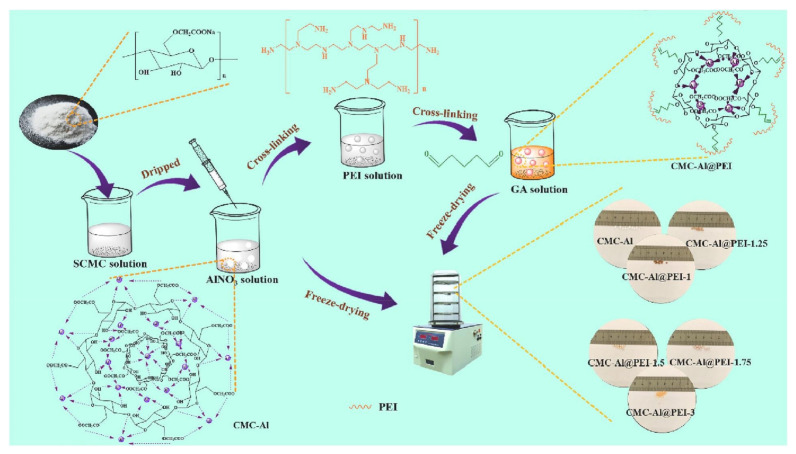
The synthesis route of the composites (Reproduced with permission from Yang et al. [53], Copyright 2021, Elsevier Ltd.).

Millimeter-size hollow microspheres were fabricated from carboxymethyl cellulose microspheres (CMC) and PEI with glutaraldehyde as a crosslinking agent [53]. The synthesis process is described in Figure 9.

The hydroxide ion in PEI in aqueous solution forms precipitate with Al(III), which can break the coordination bonds between the carboxyl and Al(III) in carboxymethyl cellulose microspheres, forming the hollow structures. SEM analysis was used to investigate the effect of usage and treatment time of PEI, and the results showed that they can make difference in shell thickness and size. The original spherical structure was completely deviated to form a powder-like structure when the PEI usage exceeded 3% and the internal network of composites were loosened as PEI treatment time increased. Thus, the adsorption of CMC-Al@PEI-1.75 was studied. The obtained hollow microsphere showed a superior performance in adsorption of MB of 235 mg/g. The large accessible amount of –NH_2_ groups and its unique hollow structure allowed the obtained microsphere to be applied in the treatment of wastewater. Another cellulose microsphere, palladium nanoparticles/cellulose microspheres (Pd NPs/CMs) were fabricated as catalyst for MB degradation [54]. The microsphere was prepared by in-situ reduction method based on the cellulosed microspheres. Pd nanoparticles grew on cellulose microspheres, with a Pd conversion rate of 33%. The spherical structure helped the cellulose microspheres function as suitable carriers for catalysts. The rate of decolorization was determined by UV-vis spectrophotometer. The decolorization efficiency of MB in presence of NaBH_4_ was over 99.8% and the microsphere showed excellent reusability for five cycles. The decolorization was conducted through the destruction of the chromophores of MB. Zeta potential was measured to investigate the effect of pH. Solutions below pH 3.5 showed a low decolorization rate. The catalytic performance was better under an alkaline condition than in an acidic condition. First, the reactants were adsorbed on the surface of the catalyst ahead of the reaction, then the ${\mathrm{BH}}_{4}^{-}\;$ transferred to metal from the surface of the catalyst. The reactions continuously relayed to destroy the chromophores of MB, consequently decolorizing it. A cellulose nanocrystal (CNC)/MnO_2_-based porous microsphere is also used for MB decolorization. It was fabricated to solve the problem of CNC-based microsphere, namely its low porosity and specific surface area that limits the adsorption ability in wastewater treatment despite its beneficial properties such as simultaneous adsorption, degradation ability, facile separation, and recyclability [55]. SEM imaging showed its porous, honeycomb-like structure which was formed by freeze-drying the air bubble templated emulsion and ionic crosslinking method that used SA as a crosslinked matrix. SA added carboxyl groups that provided negative charge for interaction with cationic MB dye. The CNC/MnO_2_/SA microsphere showed low density of 0.027 g cm^3^ and high porosity of 98.23%. The removal efficiency was measured through UV-vis spectrometry. The high porosity enhanced the adsorption effect, the decolorization of MB was conducted within 10 min up to 95.4%, and the equilibrium decolorization reached 114.5 mg/g. The great recycling ability and its green and simple fabrication process proved to be a great application prospect in the removal of dye on wastewater treatment. Three cellulose microspheres are all used for removal of MB dyes, but their removal mechanisms differ along their different functional groups.

### 3.2. Alginate

Algae that adsorb heavy metal ions are mostly used in the form of SA as adsorbents. Alginate is naturally abundant, can be obtained from seaweed, and is biodegradable and hydrophilic. It is modified in various ways; for instance, it can be mixed with zeolite and made into bead form. The efficiency of alginate as an adsorbent is summarized in Table 6.

Ferriferous oxide@hydrous cerium oxide (Fe_3_O_4_@HCO) was encapsulated into sodium alginate microbead (SAB) by an improved co-precipitation method to remove antimony (Sb(III)) ions within a concentration range of 5–60 mg/L [56]. The magnetic microsphere can be easily retrieved from aqueous solution by a magnetic separation. The introduction of SA resolves the poor precipitation and easy hardening issues of prior Fe_3_O_4_@HCO adsorbent as SA easily complexes with divalent cations, which can make gel forms. The adsorption kinetics followed the PSO model and the adsorption isotherm followed the Langmuir model, indicating that the mechanism was the result of cooperation of chemisorption (ion exchange) and physisorption (diffusion reaction). Surface hydroxyl groups of SAB whose presence was confirmed by FTIR results were the major source for the adsorption. Initial and pH and temperature had an influence on adsorption performance, and microbeads did not collapse within a pH range of 3–7. The environmentally friendly and cost-effective main components (Fe_3_O_4_ and HCO) and the facile preparation of SAB show the potential of the microsphere as adsorbent for Sb(III) in large scale.

Similarly, a hyperbranched polyamide-functionalized sodium alginate (HA@SA) microsphere was prepared for the removal of Sb(III) and followed the PSO and the Langmuir models [57]. A HA@SA microsphere was fabricated by grafting 1.0 g of HA on the surface of SA microsphere. SEM image showed that the microsphere had average diameter of 3.0 nm and surface roughness increased after the adsorption of Sb(III). The optimal adsorption was conducted at pH 5.0. and the maximum Sb(III) adsorption capacity was 195.7 mg/g, which was 1.16 times higher than SA microspheres without HA. The adsorption process was a homogeneous single-layer adsorption which was controlled by chemisorption and was an exothermic and spontaneous reaction. The HA@SA microsphere maintained its adsorption capacity of 90% after recycling for eight adsorption-desorption cycles, even at low flow rate and concentration in practical usage. A zeolite/alginate microsphere was fabricated for Ni ions removal from aqueous solution [58]. The SA microsphere improves the adsorption of zeolite adsorbent by immobilizing the zeolite powder due to its hydrophilic property. The adsorption ability of the SA-modified microsphere is supported by many characterizations. The FT-IR results shows that Zeolite particles reacted with alginate polymer through hydrogen bonding. XRD pattern show the crystalline phase is the same as that of zeolite, but in a lower intensity due to the amorphous phase. SEM micrographs shows the folds and groves of the microsphere that enhance its adsorption capacity. The microsphere showed a removal efficiency of 98% within 150 min. The kinetic study showed that the adsorption followed the PSO model, indicating that the metal ion removal rate is dependent on the number of free active sites. An isotherm study shows the sorption process follows the Freundlich model, indicating a heterogeneous surface. The Dubinin–Kaganer–Radushkevich (DKR) model study proves that removal reaction is endothermic and the sorption process is chemical. The Zeolite/alginate microspheres can be reused five times without deformation and with the same efficacy. SA can improve adsorbent in other ways; for instance, it can be used as matrix for different microsphere to produce hybrid beads. Chitosan microsphere/sodium alginate hybrid beads (CSM/SA) were fabricated for the removal of heavy metal ions from aqueous solution [59].

**Figure 10:**
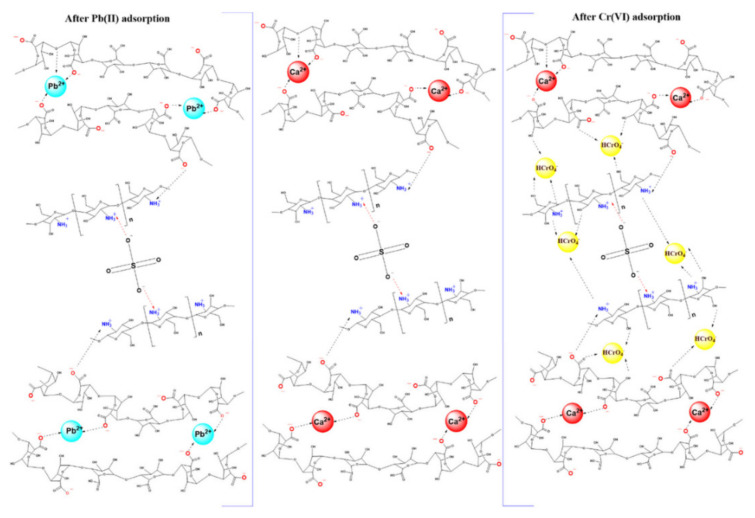
Proposed mechanism of adsorption of Cr(VI) and Pb(II) ions onto CSM/SA hybrid beads (Reproduced from Ablouh et al. [59], 2019, BioMed Central Ltd.).

The microsphere was applied in the removal of Cr(VI) and Pb(II). The properties of the hybrid beads were enhanced via mutual interaction between original chitosan microspheres and SA. Additionally, the effects of different metal concentrations, pH solutions, and contact times were evaluated. The maximum adsorption capacity obtained from atomic absorption spectrophotometer was 180 mg/g for Pb(II) and 16 mg/g for Cr(VI). EDX results confirmed that the amount of adsorbate is proportionate to the quantity of Ca^2+^ on the surface of CSM/SA hybrid beads, but Cr retention is dependent on electrostatic attraction, and the proposed mechanism is drawn in Figure 10. TGA-DTA analysis showed the high thermal stability of the CSM/SA. The first step of the four thermal degradation was from 25 to 240 °C and showed a 9.7 wt% loss. The adsorption kinetics were evaluated using the PFO and PSO models. The sorption of both Pb(II) and Cr(VI) fit the Langmuir model, indicating that it was monolayer adsorption.

A PAM/SA adsorbent with double network structure was prepared for adsorption of MB dye, while the MB concentration was determined using a UV-vis spectrophotometer [60]. The PAM/SA microsphere was prepared via emulsion polymerization of acrylamide and SA. The double network structure was formed from the entanglements between PAM and SA molecular chains. TGA results revealed that adding SA decreases the thermal stability and crystallinity of PAM, but the adsorption capacity of microsphere increased. The microsphere had a smooth surface, the particle size was in range of 1–10 μm, the pore size was in range of 0–200 nm, the mesoporous structure was uniform, and the ion crosslinking point was uniformly distributed. At pH 6, 25 °C, and 0.1 g adsorbent dosage, the maximum adsorption capacity reached 75 mg/g. –COO^−^ on SA has hydrogen bond with –OH and –NH_2_ and has electrostatic interaction with –C=N^+^ of MB, which is beneficial for MB molecule to agglomerate and be adsorbed on the surface of SA/PAM composite microspheres. The adsorption followed PFO kinetics and the Langmuir isotherm model, indicating that it was physical adsorption and that the maximum capacity was 1070.54 mg/g. The adsorption force decreased with rising temperatures. SA microspheres can also adsorb organic pollutants [61]. An N-doped reduced graphene oxide/sodium alginate/polyvinyl alcohol (NRGO/SA/PVA) microsphere was fabricated to remove anthracene (ANT), its oxygenated-polycyclic aromatic hydrocarbon (OPAH), and 2-methylanthroaquinone (2-MAQ) and reduce the risk of the application of nanomaterials in a water environment. It was prepared with 2.0 wt% SA, 1 wt% PVA, and 3.22 wt% NRGO through a simple embed method. NRGO was embedded instead of GO because it has a higher affinity for hydrophobic organic pollutants than GO. TGA and DTG analysis showed that among three steps of thermal degradation, the second stage showed the greatest weight loss of 42.7% at 275–600 °C due to the degradation of PVA and calcium alginate. The residual polycyclic aromatic hydrocarbons (PAHs) concentration was measured using a gas chromatograph-mass spectrometer. The sorption kinetics followed the PSO model and equilibrium data followed the Freundlich and D-R isotherm model, indicating that the adsorption was chemical adsorption. Hydrogen bonding and π-π interactions mainly attributed to the adsorption mechanism.

### 3.3. Chitosan

Chitosan is obtained by treating chitin, obtained from living organisms. Ascribed to its eco-friendly and abundant functional groups such as amino and hydroxyl groups, it is used as adsorbents for pollutants. Additionally, modification into magnetic microsphere is possible due to its nano particle encapsulation property. Its performances as adsorbents are summarized in Table 7.

Polyethyleneimine-functionalized chitosan/Span80 microspheres ((CYCTS/Span80)-@-PEI) were fabricated to remove diclofenac sodium (DS) from wastewater effectively [62].

**Figure 11:**
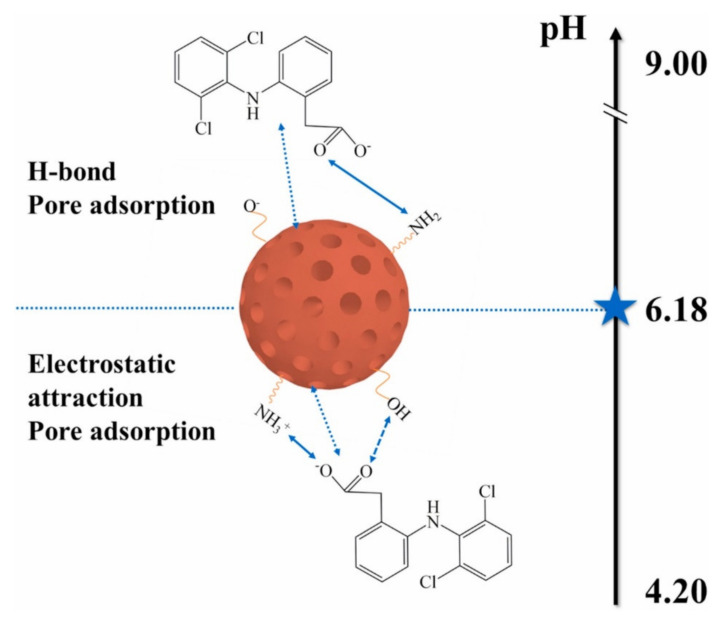
Schematic representation of the adsorption mechanism (Reproduced with permission from Jiang et al. [62], Copyright 2021, Elsevier Ltd.).

Carboxylated chitosan (CYCTS) was added dropwise into cross-linking agent Zr^4+^ solution to spherical structure. PEI was grafted on to its surface, and nonionic surfactant Span80 was dissolute from microsphere using an organic solvent for formation of pores. The surface of microsphere, examined through SEM image, had substantial number of NH^3+^ and a dense pore structure, which was useful for adsorption of DS. The DS adsorption was measured via UV-vis spectrophotometer, and the maximum adsorption was 572.28 mg/g at pH 5.00 and 27 °C with adsorbent loading of 15 mg. The adsorption process followed the PSO kinetic model, Langmuir isotherm model, and Freundlich isotherm model, indicating that the adsorption was a multi-layer process. Figure 11 depicts the main adsorption mechanisms (which were electrostatic interactions, physical adsorption, and hydrogen bonding). The removal rate of DS remained over 80% after five cycles with a high recovery rate.

**Figure 12:**
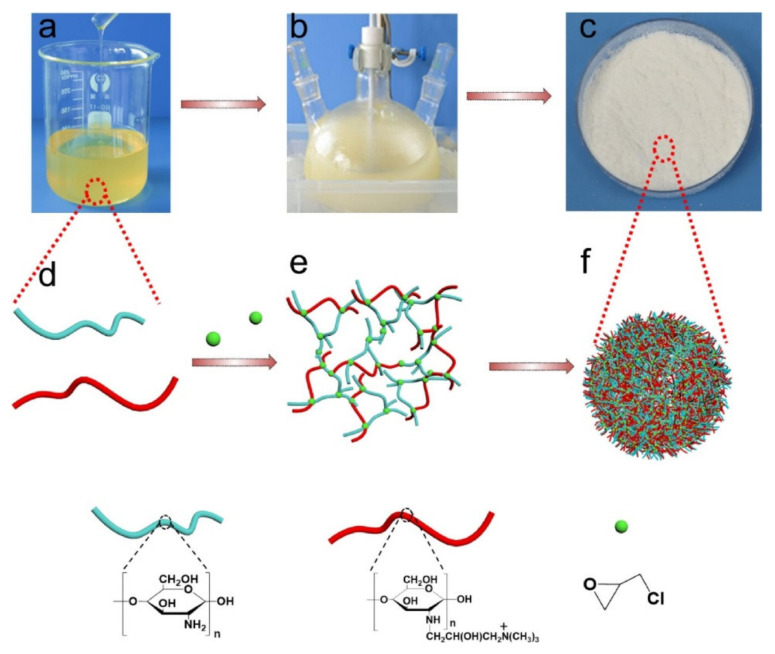
Schematic diagram for the fabrication process of CCQM, the dissolved mixed solution of chitosan and quaternized chitosan (**a**) the corresponding simulated image (**d**), photograph of cationic polyelectrolyte microsphere preparation (**b**), the simulated cross-linked network image of chitosan and quaternized chitosan (**e**), the photograph of dried CCQM (**c**) and the corresponding simulated image (**f**) (Reproduced with permission from Cai et al. [65], Copyright 2021, Elsevier Ltd.).

A chitosan-bismuth cobalt selenide hybrid microsphere (BSCM-CM) was made to remove organic pollutants from aqueous environment [63]. Bismuth cobalt selenide (BSCN) tri-composite nanoparticles with crystalline structures and a narrow bandgap of 2.48 eV were prepared and were embedded in chitosan microspheres via a solvothermal process to prevent the leaching of the photocatalyst and to enhance the recovery and reuse of the composite. The SEM image, FT-IR, and XRD results confirmed that the obtained BSCM-CM had smooth face morphology with an average nanoparticle size of 30 nm, an average microsphere size of 734 μm, and a crystallite size of 21.3 nm. In sunlight radiation, the degradation efficiency for CR dye was 85% of the 90 ppm solution at catalyst amount of 0.6 g, pH 8, for 100 min. The adsorption was well described by first order kinetics, with a constant rate of 1.50 × 10^−2^ min^−1^. The microsphere could be regenerated and reused for up to five cycles. Similarly, other CMs with BiSe, zinc-bismuth-selenide-chitosan microspheres (ZBiSe-CM), were fabricated as catalysts for photocatalytic degradation of CR dye [64]. First, ZBiSe nanoparticles were synthesized via a solvothermal process and then crosslinked with chitosan for support and to prevent leaching of the catalyst. SEM imaging showed that the average size was 30.9 nm for each nanoparticle and 812 μm for the spherical and porous microsphere, and XRD analysis confirmed that crystallite size was 27.04 nm for the nanoparticles. The degradation efficiency of the ZBiSe-NP reached 99.63% for 40 ppm concentration of CR at catalyst dosage 0.225 g, pH 8.0, and 36–38 °C for 2 h of solar light illumination. The photodegradation of microsphere followed PFO kinetics, and the microsphere remained its high decontamination efficiency for five cycles. Both BSCM-CM and ZBiSe-CM shows high potential for photocatalytic degradation of CR dye using clean and renewable energy of solar light irradiation sources, while ZBiSe-CM showed higher degradation efficiency than BSCM-CM. The effects of several factors for the absorption and the relationship between the factors were investigated using response surface methodology (RSM) modeling. A quaternized chitosan@chitosan cationic polyelectrolyte antibacterial microsphere (CCQM) was fabricated via the chemical crosslinking and emulsification method for removal of dyes and heavy metal ions [65]. Figure 12 presents the fabrication process.

CCQM has a higher specific surface area, pore volume, and surface charges, lower crystallinity, and displayed higher adsorption capacities than did pristine chitosan microspheres. Through DTG analysis, it was found that the highest decomposition rate was at a lower temperature than pristine chitosan and that the addition of quaternized chitosan increased the thermal stability. The adsorption followed the Langmuir and PSO models and Fickian diffusion laws’ equation, indicating that the adsorption was primarily monolayer chemisorption and pore diffusion mainly determined the adsorption rate. The adsorption reached equilibrium within 4 min, and the adsorption capacity was 1500 mg/g for CR, 179.4 mg/g for MO, and 687.6 mg/g for Cu(II), and 398.8 mg/g for Fe(III). The effective absorbance of Cu^2+^ is due to its smaller steric hindrance. Cation exchange, chelating effect, proton exchange, and complex formation facilitated effective heavy metal ion removal, and electrostatic interaction and hydrogen bonding facilitated the removal of dyes. CCQM can be used as a filler in adsorption columns to purify wastewater, as it has superb recyclability, antibacterial viability for sterilizing, and biodegradability within 165 days. Other CMs made using the emulsion cross-linking method is chitosan-activated carbon composite microspheres, which were prepared for adsorption of MO [66]. A chitosan solution was mixed with activated carbon powder and then chitosan was cross-linked by epichlorohydrin under microwave irradiation, which effectively shortens the cross-linking time. SEM imaging displayed that the obtained microsphere had a diameter of 200–400 μm and activated carbon powder was dispersed on the surface of chitosan composite microsphere. The maximum adsorption of MO was obtained when the mass ratio of chitosan to activated carbon was 10:4, labeled CA4, and the capacity was 35.4 mg/g. TG curves showed that CA4 loses weight rapidly in the temperature range 200–350 °C due to the decomposition of chitosan. The adsorption was dependent on temperature, and maximum adsorption occurred at 30 °C. The adsorption followed the PSO model and the Freundlich isotherm model. The microsphere retained 94.49% of its adsorption capacity for MO after it was reused for three cycles. A quaternary ammonium salt-modified chitosan microsphere (CTA-CSM) was prepared for the treatment of dye in wastewater [67]. The CTA-CSM was fabricated through an emulsion cross-linking reaction between 3-chloro-2-hydroxypropyl trimethyl ammonium chloride (CTA) and chitosan. SEM imaging confirmed that the microsphere had smooth surface and was well dispersed. Through laser light scattering particle size analyzer, it was discovered that particle size was distributed in the range of 5 to 125 μm. The grafting of CTA on the CS microsphere enhanced the adsorption capacity for MO dye from previous reports, as the maximum removal rate was 98.8% and adsorption capacity was 131.9 mg/g under optimal condition. The MO adsorption process well fitted by the Langmuir isotherm model and followed the PSO kinetic model. Additionally, the CTA-CSM retained its high removal rate after it was reused for five cycles over 87.4%. Thus, CTA-CSM is a promising adsorbent for treating wastewater that contains MO dye for its cost-effective, sustainable, and highly reusable properties. For the removal of cationic dyes and MB, a MCS-g-PSSS microsphere was made by grafting poly(sodium 4-styrene sulfonate) onto magnetic chitosan microspheres [68].

**Figure 13:**
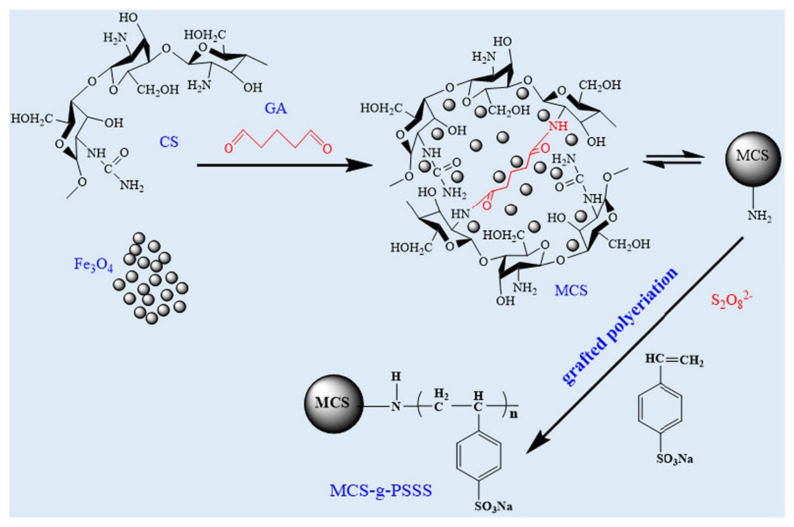
The schematic diagram of preparing MCS-g-PSSS microspheres (Reproduced with permission from Men et al. [68], Copyright 2021, Elsevier Ltd.).

Figure 13 shows the preparation process. The magnetic chitosan microspheres were prepared by reverse-phase suspension. Then, SSS was grafted onto MCS microspheres. XRD patterns showed that MCS-g-PSSS microsphere still had Fe_3_O_4_ peaks, which implies that the microsphere is still magnetic after grafting of PSSS. Magnetic hysteresis loops show that the saturation magnetization the microsphere was 5.2 emu/g. The adsorption process followed PSO model, and the adsorption isotherms followed the Langmuir model. The saturated adsorption capacity reached 854 mg/g for MB at an initial concentration of 1000 mg/L at pH 7.0 and 0.02 g of feed dosage at 25 °C. FT-IR and XPS results confirmed that the high adsorption capacity is driven from the strong electrostatic interactions and π-π stacking. The adsorption capacity increased with a rising temperature and decreased with ion addition. The spontaneity of the adsorption increased as temperature increased.

Chitosan microspheres are also used as adsorbents of heavy metal ions. A porous chitosan microsphere was produced without other polymers using the freezing-lyophilization drying method for the removal of hexavalent chromium, Cr(VI) [69]. The optimal chitosan microsphere was produced by freezing chitosan hydrogel beads at −20 °C and subsequently lyophilizing the frozen structure, which allowed for the easy obtaining of some beneficial structures of the chitosan microspheres for efficient removal, rough surfaces, and large pores. The microsphere was characterized using porosity analysis. Microspheres with good sphericity, thinner pore walls, and small pore sizes made with an initial chitosan solution concentration of 3% (*w*/*v*), a syringe diameter of 500 μm, a pH range from 3.0 to 5.0, a temperature of −20 °C, and 72 h freezing time showed a maximum adsorption capacity of 945.2 mg/g for Cr(VI). The adsorption capacity increased as freezing time increased. The adsorption rate was governed by multiple steps, and the adsorption was primarily attributed to amine and hydroxy groups of chitosan microspheres. In optimal conditions, the equilibrium adsorption rate was 1.83 × 10^−5^ g/mg·min and the adsorption amount at equilibrium was 107.05 mg/g. Cross-linking of CS improves mechanical property, but it consumes the functional groups that are beneficial for adsorption. Thus, a chitosan-methyl acrylate-diethylenetriamine microsphere was made without any cross-linking agent to uptake Pb(II) and Cd(II), from wastewater [70].

**Figure 14:**
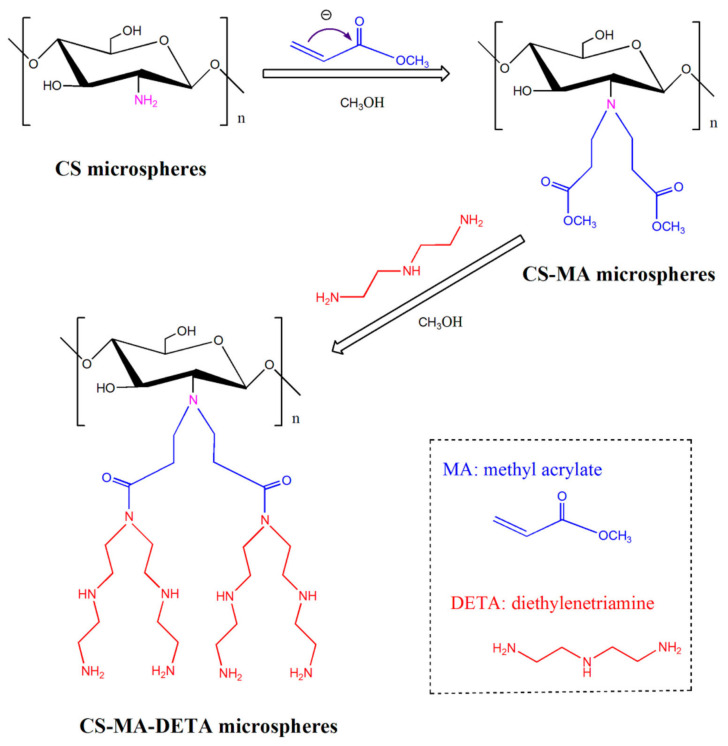
Schematic Preparation Procedures of CS-MA and CS-MA-DETA Microspheres. (CS-MA-DETA, DETA Grafted CSMA; DETA, Diethylenetriamine; CS-MA, MA Grafted CS; MA, Methyl Acrylate; CS, Chitosan.) (Reproduced with permission from Zhang et al. [70], Copyright 2017, American Chemical Society).

The CS-MA-DETA was fabricated using two steps as shown in Figure 14. First, MA was grafted to amino groups on CS microsphere surface. Next, DETA was grafted to terminal ester groups of MA on CS-MA microsphere surface. The fabrication was conducted without cross-linker, confirmed by FTIR and elemental analysis.

**Figure 15:**
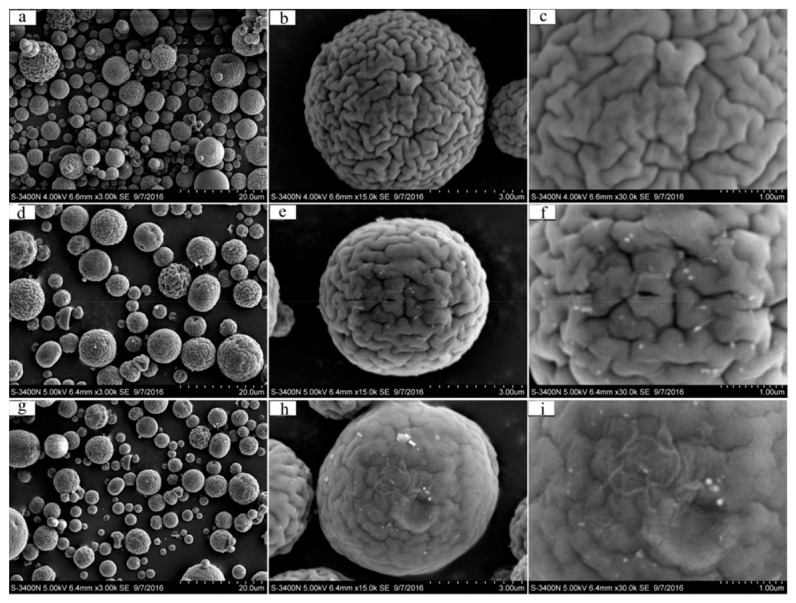
SEM images of CS, CS-MA-DETA, and Pb(II)-loaded CS-MA-DETA microspheres. ((**a**–**c**): pictures of CS microspheres; (**d**–**f**): pictures of CS-MA-DETA microspheres; (**g**–**i**): pictures of CS-MA-DETA microspheres after five-time adsorption for Pb(II); pictures of each group above are in turn taken at magnifications of 3 k, 15 k, and 30 k×. CS-MA-DETA, DETA grafted CS-MA; DETA, diethylenetriamine; CS-MA, MA grafted CS; MA, methyl acrylate; CS, chitosan.). (Reproduced with permission from Zhang et al. [70], Copyright 2017, American Chemical Society).

The SEM image in Figure 15 shows that the microsphere had 3.04 μm mean diameter and had uniform wrinkle-like topography, which are beneficial to capture metal ions. Elemental analysis showed that addition of MA increases the C/N ratio to 4.76. The specific surface area was 27.806 m^2^/g, the pore diameter was 3.452 nm, and TGA curves revealed that mass loss at the first step was 3%, which is around 10% of CS. Both CM with PHEMA brushes and CS-MA-DETA microsphere followed the PSO model and the Langmuir model, indicating that the adsorption process was primarily monolayer adsorption and chemisorption.

**Figure 16:**
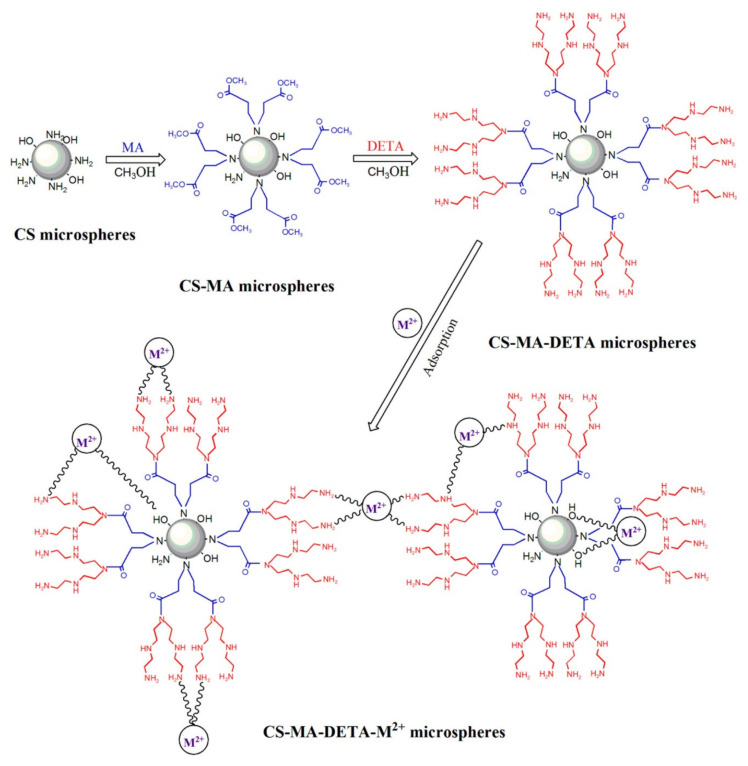
Schematic preparation procedure of CS-MA-DETA microspheres and probable bonding modes and adsorption sites for Cd(II) and Pb(II) on CS-MA-DETA microsphere surface. (CS-MA-DETA, DETA grafted CS-MA; DETA, diethylenetriamine; CS-MA, MA grafted CS; MA, methyl acrylate; CS, chitosan.) (Reproduced with permission from Zhang et al. [70], Copyright 2017, American Chemical Society).

Figure 16 shows the possible bonding modes and adsorption sites. The efficiency was 239.2 mg Pb(II)/g, or 201.6 mg Cd(II)/g. The microsphere can be readily regenerated and recycled for five cycles. Cross-linked chitosan (CCS) microspheres tethered with melamine-conjugated poly(hydroxyl methacrylate) (PHEMA) brushes were synthesized for effective Cu(II) uptake [71]. In order to solve the issue of consuming functional groups while cross linking, PHEMA and melamine was incorporated to add functional groups in this case. The microsphere was fabricated via surface-initiated atom transfer radical polymerization (ATRP) of hydroxyethyl methacrylate (HEMA) along with melamine being subsequently covalent immobilized onto the ends of the poly(hydroxyethyl methacrylate (PHEMA) brushes’ chain. The water contact angle was measured to investigate the surface wettability. Grafting PHEMA brushes lowers the water contact angle due to the abundant hydroxyl groups of the surface of microsphere. The sufficient nitrogen from triazine heterocyclic structures and oxygen from the PHEMA chains offered increased adsorption sites. Adsorption of Cu(II) reached equilibrium within 20 min, and the adsorption process was controlled by intra-particle diffusion and chemisorption processes. The maximum adsorption capacity was around 4.68 mmol/L (or 299 mg/g) at pH 5, 55 °C and an initial Cu(II) concentration of 2.21 mmol/L. The adsorption mechanism was mainly ascribed to coordination or chelation interactions of amino or hydroxyl groups with Cu(II) ions.

## 4. Photocatalytic Degradation

An advanced oxidation process (AOP) that involves the production of ·OH is one promising method for removal of organic pollutants [72]. In one method of AOP, Fenton process, ·OH is easily produced by Fe^2+^ catalyst and H_2_O_2_ is often used as one method, but the conventional method requires large amounts of iron sludge and a high dosage of H_2_O_2_ [73]. The combination of heterogenous photo-Fenton and photocatalysis enhances the degradation efficiency of pollutants as well as recyclability and overcomes many problems that conventional methods face [74]. Introducing polymer such as chitosan and PVA can enhance the performance of catalyst through enhanced mechanical properties and adsorption capacity [75]. Immobilization of photocatalysts such as ZnO or TiO_2_ can help inhibit the recombination of electrons and holes, increasing ·OH production. Such modifications have the ability to scale up for commercial uses [76]. Table 8 summarizes the efficiency of photocatalytic spheres.

The photo-Fenton process is also used in the decolorization of dyes. Fe/polymer-based photocatalysts were made for the adsorption of MB [77]. Alginate and chitosan spheres were studied, but since the polymer chain of alginate chitosan degraded under UV irradiation, only the chitosan sphere was investigated further.

**Figure 17:**
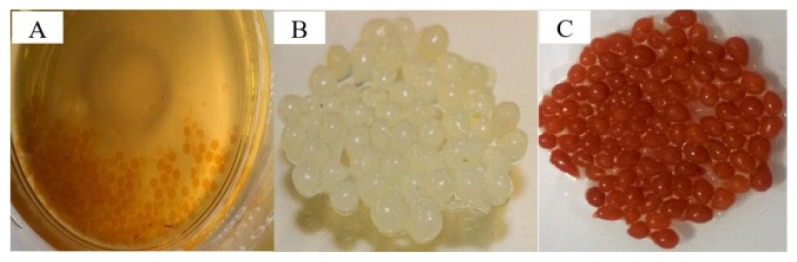
Fe/Alg beads after 30 min of photo-Fenton reaction (**A**), reticulated chitosan spheres (**B**) and reticulated chitosan spheres after iron sorption (**C**) (Reproduced with permission from Lopes et al. [77], Copyright 2020, Elsevier).

Figure 17 shows the Fe/Alginate beads and chitosan spheres. Immobilization of Fe^2+^ was conducted via ultrasound (US) bath and EDS analysis confirmed that the sorption of iron process took 15 min, substantially shorter than the conventional way, which takes 30 h. The amino group of chitosan captures the Fe^2+^ ions. The removal efficiency for MB reached 98.8% within 6 min at pH 3, with 300 mg/L of H_2_O_2_ and 2 g of Fe/chitosan/US-15 min sphere. The effects of such factors were investigated using RSM. TG, DTG analysis showed that the main decomposition occurred at 150–350 °C. The discoloration followed the PFO kinetic model and the L-H isotherm model. For actual industrial wastewater, the catalytic sphere showed 96% for decolorization and a very high efficiency toward real effluents. Another photocatalyst for dye removal is FCF/PDA-PVDF beads (FDPB), which were fabricated by carbothermal reduction to immobilize Fe species/biochar fiber composite (FCF) catalyst for photodegradation of MO [78]. Powdered FCF was mixed with PDA-modified (polyvinylidene fluoride) PVDF through a phase inversion process. SEM imaging shows that FDPB had macropores varying in the range of 1.0 μm to 100 μm inside the beads. The structure allows the self-floating property derived from abundant air chambers inside the bead, which is beneficial for collecting light for photodegradation. Electron-rich material such as FCF increases the reduction of Fe^3+^ into Fe^2+^. In addition, PDA excited under visible light can produce electrons for formation of ·OH.

**Figure 18:**
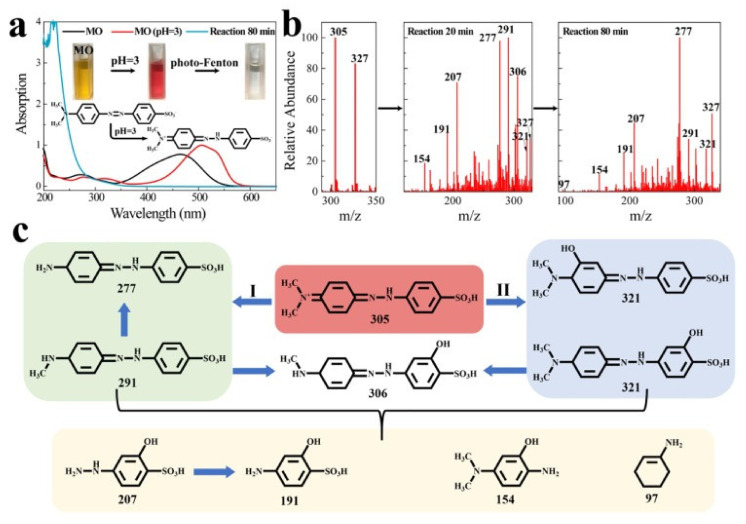
UV–vis spectra (**a**), GC–MS (**b**) analysis and possible degradation pathways (**c**) of degrading MO during photo-Fenton reaction (Reproduced with permission from Li et al. [78], Copyright 2020, Elsevier).

Figure 18 shows result of MO degradation by the UV-vis spectra and assumed degradation pathways via LC-MS analysis. The FDPB almost showed perfect decolorization efficiency in 60 min. The adsorption mechanism was described in two stages, surface adsorption and intra-particle diffusion.

**Figure 19:**
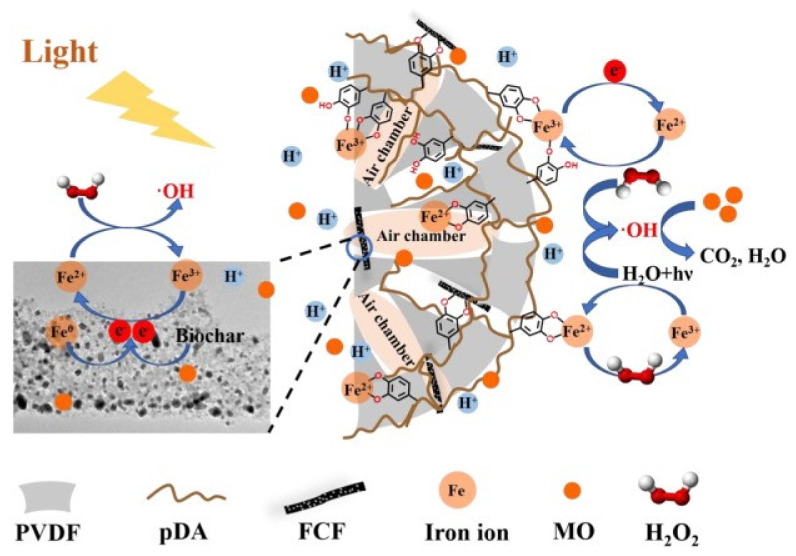
Possible mechanism diagram for degrading MO over FDPB in photo-Fenton system (Reproduced with permission from Li et al. [78], Copyright 2020, Elsevier).

Figure 19 shows the schematic of the assumed mechanism of photo-Fenton degradation. FDPB has a hierarchical porous structure that allows for the rapid diffusion of MO, and PDA does its role of blocking the leaching of iron by its abundant catechol groups and by enhancing the adsorption and diffusion of MO.

### 4.1. Alginate

Alginate beads are often used as a network for photocatalysts. Light can readily penetrate through an alginate bead from outside of the bead. It can be used as photo-bioreactor removal of nutrients (not just dyes) from wastewater [79]. PVA/SA hydrogel beads were fabricated with a across-linking agent of FeCl_3_ and boric acid as the catalyst for the photo-Fenton process of TC [80]. Hydrogels rose as a promising 3D material as a heterogenous photo-Fenton catalyst. The optical image of PVA/SA-FeCl_3_ bead showed that it had a diameter around 5 mm, and SEM imaging showed that average pore size was around 1.16 μm. The removal rate reached a maximum of 90.5% within 60 min for TC, at an H_2_O_2_ concentration of 6 mM under visible-light irradiation. The degradation increased when H_2_O_2_ concentration increased from 2 to 6 mM, but decreased after it reached 6 mM, which may be attributed to excessive secondary reactive species that can compete with reactive species for photocatalysis such as ·OH or h^+^. While the TC reached 90.5%, the degradation efficiency was 28.6% for TOC. Through free-radical and hole-trapping experiments, the main species for TC degradation was revealed as h^+^, with ·O_2_^–^ and ·OH also contributing. Inductively coupled plasma atomic emission spectroscopy (ICP measurement) results suggest that the PVA/SA-FeCl_3_ beads have good stability through repeated degradation cycle, but after five cycles, Fe could leach out from the catalyst, therefore requiring an enhancement in stability. Other catalysts for dye removal were made using HAP. Three catalysts were made from SA beads, SA-magnetic HAP (Alg-mHAP), Alg-m, Alg-HAP, for removal MO [81]. HAP supports and strengthens the catalyst performance due to its high ion-exchange and adsorption capacity. SEM imaging showed that the morphology of sphere SA catalysts changed after the introduction of HAP, Fe_3_O_4_ in that the surface became rougher. FT-IR spectra indicate that the phosphate group in HAP and the carboxyl group in alginate are the functional groups, and they remain stable through the photo-Fenton process. Those non-ionized functional groups limit the catalysis to operating in acidic pH in order to minimize the electrostatic repulsion between the anionic MO and the catalyst. The Alg-mHAP catalyst showed the best discoloration among three, 84.28% within 90 min at pH 3, which was the optimal pH. In same condition, Alg-m reached 79.41%, and Alg-HAP reached 71.42%. The removal rate increased as H_2_O_2_ concentration increased from 0.5 to 3 mM; however, the rate decreased over 3 mM. The MO degradation fit first-order kinetic model for all three catalysts. Aside from pure SA beads, SA can be modified with other bivalent cations such as Ca^2+^, Ba^2+^, and Zn^2+^ for structural change. BiOCl-calcium alginate (BOC-CA) hydrogel beads were fabricated for the removal of RhB dye [82]. CA can be fabricated by cross-linking Ca^2+^ with SA. The cross-linked structure has an “egg-box”-like 3D network. BiOCl, which is used as photocatalyst in BOC-CA, can produce oxygen vacancies (OVs) on the surface under UV irradiation, providing a path for the transport of photogenerated carriers. OVs prevent the recombination of holes and electrons by serving as centers for capturing electrons. SEM and optical microsphere images show that BOC-CA has spherical and porous structure, which allow the beads to float on the surface of water, a beneficial property for the absorbance of light. The decolorization efficiency reached 100% within 75 min under UV light, and 90 min under visible light. The efficiency was even high after reuse.

**Figure 20:**
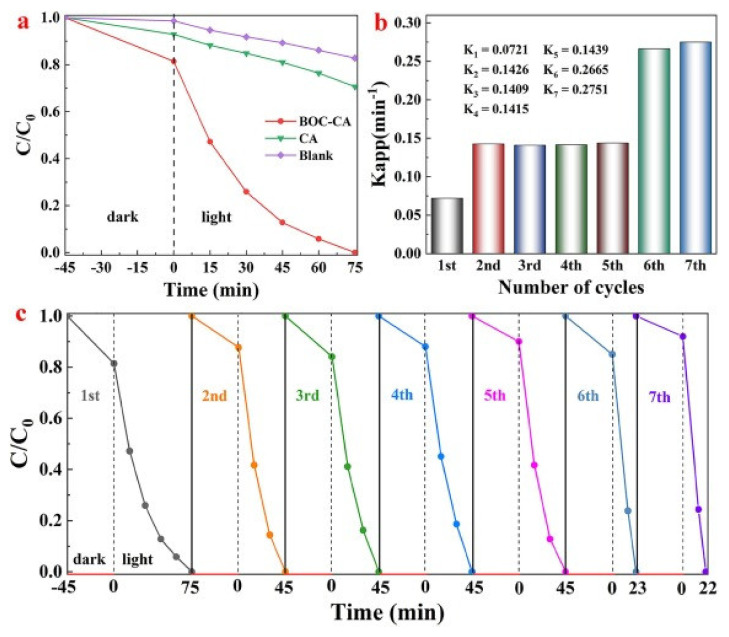
(**a**) UV light irradiated photocatalytic degradation curves, (**b**) the corresponding K parameters in the presence of 2 wt% BOC-CA beads and (**c**) cyclic degradation curves of RhB (Reproduced with permission from Liong et al. [82], Copyright 2021, Elsevier).

As Figure 20 shows, the complete decolorization was achieved within even shorter time as it was repeatedly used. The degradation rate increases with increasing cycle, but the mass of the bead itself decreases; however, the BOC-CA bead still shows a high stability than the pure CA bead.

### 4.2. Chitosan

Photodegradation is also used for the removal of organic pollutants besides dyes. Chitosan beads can provide protection for nanoparticle catalysts that are highly reactive in air or aqueous solution. Chitosan beads modified with Tiourea-Magnetite-TiO_2_ (Cs-T-M-Ti) beads were prepared for the adsorption of Naphthalene [83]. However, it did not present significant differences from the Cs-T model. Therefore, converting it as photocatalyst, making use of TiO_2_, one of the most popular substances used as photocatalyst, is suggested. ZnFe_2_O_4_-CS beads were fabricated for the removal of chlordimeform insecticide (CDM) from wastewater [84]. The beads were made using the alkaline coprecipitation method. The bead had a saturation magnetization intensity of 6.42 emu/g and a coercivity of 0.93 mT. Overall, 1 g of ZnFe_2_O_4_-CS beads reached complete degradation for CDM within 7 min at optimal conditions, pH 3, an initial CDM dosage of 20 mg/L, and a molar ratio of Zn/Fe at 0.35. The removal of complete TOC removal required 2 h. H_2_O_2_ concentration could shorten the time for TOC removal, as over 80% of removal was possible within 1 h at H_2_O_2_ concentration of 8.8 mM. The catalytic degradation for CDM is majorly attributed to the activation of H_2_O_2_ under UVC irradiation. The ZnFe_2_O_4_-CS beads catalyst could be reused for at least four cycles with small metal leaching. However, the treatment for real wastewater did not always present perfect results. Chitosan beads were used as solid catalysts for the photo-Fenton process [85]. The beads were crosslinked with glutaraldehyde and mixed with FeSO_4_·H_2_O to obtain the iron-adsorbed chitosan beads. The degradation experiment treated the biological treatment effluent (E2). The effluent carried strong purple coloring, and the chitosan bead aimed to decolorize and remove the toxins. The degradation of color achieved 91.91% after 105 min and the degradation of aromatic compound reached 70.87% indicating that those compounds were fragmented into smaller compounds. However, the total dissolved solids (TDS) concentration remained at 5993.88 mg/L, which was only a 12.70% reduction. The D. magna toxicity for E2 after treatment for 105 min was presented by the effective concentration that affects 50% of the population of the organisms (EC50), EC50 increased from 63.12% to 76.57%, indicating that the toxicity decreased but the effluent remained slightly toxic. The result did not satisfy the required standard for toxicity test. It indicates that even though the decolorization was successful, this does not always imply the successful removal of toxic substances. Intermediates could be made through the removal process that may carry toxicity.

## 5. Conclusions and Future Prospects

Polymer microspheres are rising candidates as adsorbents for wastewater treatment. The removal of pollutants is a critical issue because they can cause severe health problems and drinking water shortages. MBs are extensively used in industrial procedures including textile, manufacturing, and bio industries in aquatic conditions. Their toxicity can cause cancer, mutation, and many other diseases. Therefore, the decolorization of dyes and the removal of other pollutants such as heavy metal ions, organic pollutants, and oil are urgent issues. Among several ways to remove the pollutants, adsorption is effective because it is relatively cost-effective, can operate in mild conditions, and does not produce secondary pollution.

Currently, activated carbon is widely used as the adsorbent, but due to its high cost, research to substitute AC is ongoing. The next adsorbents require chemical and mechanical stability, high recyclability, cost-effectiveness, eco-friendliness, non-toxicity, effective performance at low pollutant concentration, the ability to scale up for commercial use, and so on. To increase the adsorption capacity, abundant adsorption sites and high selectivity for the adsorbate are needed as well. We reviewed various research that used polymer microsphere as adsorbents. Synthetic polymers (including vinylic polymers and PDA polymers) and natural polymers (including cellulose, algae, and chitosan) were used. Some were made into a magnetic microsphere and photocatalyst. Most were conducted in batch experiments with UV-vis spectroscopy to measure the adsorption capacity. The optimal conditions for adsorption, recyclability, and regeneration ratio were also measured to evaluate effectiveness. RSM modeling was used in some research to find the influence of several factors such as pH, time, temperature, pollutant concentration, etc. Adsorption mechanisms, isotherm, and kinetic models were investigated.

Magnetic adsorbents can be made with various polymer microspheres by introducing magnetic nanoparticles. They showed potential for ecological adsorbents. Magnetic adsorbents have high recyclability due to their easy separation. Magnetic separation is a facile way to recover the adsorbent after it reaches equilibrium. Adsorbents can be easily recovered if an external magnetic field is given. Therefore, it can be used for several adsorption-desorption cycles. Additionally, because it is easily retrieved from wastewater, it does not drive secondary pollution from the remaining adsorbents. The polymer microsphere strengthens magnetic adsorbent by increasing adsorption capacity and stability.

It is suggested that polymer microspheres are useful adsorbents for wastewater treatment, but some further studies are required to upscale the adsorbents for broader uses and to increase the capacity even at very low pollutant concentration. Investigation toward real effluents should be done to meet actual industrial and commercial uses. Selective and efficient adsorption even in ambient or mild operating conditions are required. In addition, the decolorization of dyes does not necessarily mean the removal of toxicants. Intermediate compounds that still carry toxicity could be produced during the removal process. Therefore, further studies are still required to investigate possible intermediate compounds.

## Figures and Tables

**Table 1:** Summary efficiency of polymer as adsorbent.

S. No	Polymer	Adsorbate	Results	Kinetic Model	Isotherm Model	Reference
1	Sodium silicate	Pb(II)	629.21 mg/g	PSO	Langmuir, D-R	[24]
2	HGM@MPD-ALS	AG25, BF dyes	454.55 mg/g, 588.24 mg/g	PSO	Langmuir	[25]
3	β-CD-GO aerogel	MB, RhB, AR87, MO dyes, 2,4-DCP, PRO, EO, 4BPA	439, 388, 234, 167, 17, 49, 19, 38 mg/g	-	-	[26]
4	CG/KGP	MB dye	24.6 mg/g	PSO	Langmuir	[27]
5	PMMA@Fe_3_O_4_@DR	RhB, ST dye	99%, 98%			[28]
6	PAM	MB, NR, GV dye	1990, 1937, 1850 mg/g		Langmuir	[29]
7	MF resin	PFOA	1.18 mM/g	PSO	Freundlich, Langmuir	[30]
8	Poly[ANE + N-PMI]-TiO_2_	RhB, TC dye	95% for 50 mg/L RhB, 97% for 100 mg/L TC	PFO	L-H	[31]
9	SiO_2_/PANI/BiOBr	Oil-water emulsion separation	TOC content < 5 mg/L Flux recovery ratio 99.8%	-	-	[32]

PSO: pseudo-second-order, D-R: Dubinin–Radushkevich, HGM: hollow glass microsphere, MPD: m-phenylenediamine, ALS: sodium allyl sulfonate, AG25: acid green 25, BF: basic fuchsin, β-CD: β-cyclodextrin, GO: graphene oxide, MB: methylene blue, RhB: rhodamine B, AR87: acid red 87, MO: methyl orange, 2,4-DCP: 2,4-dichlorophenol, PRO: propranolol hydrochloride, EO: contraceptive ethynyl estradiol, BPA: bisphenol A, CG: coal gangue, KGP: geopolymer, PMMA: poly(methyl methacrylate), DR: diazo-resin, ST: safranin T, PAM: polyacrylamide, MF: melamine formaldehyde, NR: neutral red, GV: gentian violet, PFOA: perfluorooctanoic acid, ANE: trans-anethole, N-PMI: N-Phenylmaleimide, TC: tetracycline, PFO: pseudo-first-order, L-H: Langmuir–Hinshelwood, PANI: polyaniline, TOC: total organic carbon.

**Table 2:** Summary efficiency of vinylic polymer as adsorbent.

S. No	Polymer	Adsorbate	Result	Kinetic Model	Isotherm Model	Reference
1	PVA	CODMn removal rate	16.99%	-	-	[33]
2	poly(EGMDA-VIM)	phenol	34.7441 mg/g	PSO	Freundlich	[34]
3	PAN, phenolic resin	oil removal efficiency	93.6%	-	-	[35]
4	P(St-DVB)/CuNi	Pb(II), Cd(II), Mn(II), Zn(II)	15.60, 5.28, 22.42, 20.57 mg/g	PFO	Langmuir	[36]
5	St-DBV-SH	Cu(II), Zn(II), Cd(II), Pb(II), Ni(II)	45.26, 32.42, 62.77, 135.85, 49.88 mg/g	PSO	Langmuir	[37]
6	SPS	Pb(II), Zn(II), Cu(II)	49.16, 15.38, 13.89 mg/g	PSO	Langmuir	[38]
7	DVB, TEVS	nitrobenzene, 4-nitrophenol, phenol	-	-	-	[39]
8	P(VP-DVB)-6	SY, BPB dye	261.78, 277.01 mg/g	PSO	Langmuir	[40]

PVA: polyvinyl alcohol, CODMn: chemical oxygen demand using KMnO_4_, EGDMA-VIM: ethylene glycol methacrylate-n-vinyl imidazole, PAN: polyacrylonitrile, St-DVB: styrene-divinylbenzene, SPS: sulphonated polystyrene, TEVS: triethoxyvinylsilane, VP: vinyl pyridine, SY: sunset yellow, BPB: bromophenol blue.

**Table 3:** Summary efficiency of PDA as adsorbent.

S. No	Polymer	Adsorbate	Adsorbent Capacity	Kinetic Model	Isotherm Model	Reference
1	PDA	MB dye	191.55 mg/g	PSO	Langmuir, Temkin	[41]
2	PDA	MB dye	90.7 mg/g	PSO	Langmuir	[42]
3	PDA	MB, MG, NR dye	93.86, 91.98, 88.58 mg/g	PSO	Langmuir	[43]
4	PAM/PA/PDA	Cu(II)	231.36 mg/g	-	-	[44]
5	PDA	Cr(VI)	199.6 mg/g	PFO	Langmuir	[45]
6	PDA@ZIF-8	Cr(VI)	136.56 mg/g	PSO	Langmuir	[46]

PDA: polydopamine, MG: malachite green, PA: phytic acid, ZIF-8: zeolitic imidazolate framework-8.

**Table 4:** Summary efficiency of natural polymer as adsorbent.

S. No	Polymer	Adsorbate	Adsorbent Capacity	Kinetic Model	Isotherm Model	Reference
1	Lignin	Lignin extraction efficiency	21.62 g/L	-	-	[47]
2	PVMA	Cu(II)	135 mg/g	PSO	Freundlich	[48]
3	HAP	U(VI)	2659 mg/g	PSO	Freundlich	[49]
4	NH_2_-SA/PNIPA	Cu(II), Cd(II)	57.5 mg/g, 100.5 mg/g	PSO	Langmuir	[50]
5	PEI/HMPCR	Cd(II)	143.6 mg/g	PSO	Langmuir	[51]
6	PAM, MBA, pine pollen	MB, MV dye	668.96, 749.69 mg/g	PSO	Langmuir	[52]

PVMA: poly (vanillin methacrylate), HAP: hydroxyapatite, SA: sodium alginate, PNIPA: poly-N-isopropyl acrylamide, PEI: polyethyleneimine, HMPCR: cassava residue, MBA: N-N’-methylene-bis-acrylamide, MV: methyl violet.

**Table 5:** Summary efficiency of cellulose as adsorbent.

S. No	Polymer	Adsorbate	Adsorbent Capacity	Functional Group	Reference
1	CMC-Al@PEI-1.75	MB dye	235 mg/g	Amino	[53]
2	Pd NP/cellulose	MB dye	99.8%	Hydroxyl	[54]
3	CNC/MnO_2_/SA	MB dye	95.4%, 114 mg/g	Carboxyl	[55]

CMC: carboxymethyl cellulose, NP: nano particle, CNC: cellulose nanocrystal.

**Table 6:** Summary efficiency of alginate as adsorbent.

S. No	Polymer	Adsorbate	Adsorbent Capacity	Kinetic Model	Isotherm Model	Reference
1	Fe_3_O_4_@HCO in SA microbead	Sb(III)	15.368 mg/g	PSO	Langmuir	[56]
2	HA@SA	Sb(III)	195.7 mg/g	PSO	Langmuir	[57]
3	zeolite/alginate	Ni(II)	98%	PFO	Freundlich	[58]
4	CSM/SA hybrid bead	Pb(II), Cr(VI)	189, 16 mg/g	PFO, PSO	Langmuir	[59]
5	PAM/SA	MB dye	1070.54 mg/g	PFO	Langmuir	[60]
6	NRGO/SA/PVA	ANT, 2-MAQ	0.72, 0.70 mg/g	PSO	Freundlich, D-R	[61]

HCO: hydrous cerium oxide, HA: hyperbranched polyamide, CM, CSM: chitosan microsphere, NRGO: N-doped reduced graphene oxide, ANT: anthracene, 2-MAQ: 2-methyllanthraquinone, D-R: Dubinin–Radushkevich.

**Table 7:** Summary efficiency of chitosan as adsorbent.

S. No	Polymer	Adsorbate	Adsorbent Capacity	Kinetic Model	Isotherm Model	Reference
1	(CYCTS/Span80)-@PEI	DS	572.28 mg/g	PSO	Langmuir, Freundlich	[62]
2	BSCM-CM	CR dye degradation efficiency	85%	first order	-	[63]
3	ZBiSe-CM	CR dye degradation efficiency	99.63%	PFO	-	[64]
4	CCQM	CR, MO dye, Cu(II), Fe(II)	1500, 179.4, 687.6, 398.8 5 mg/g	PSO	Langmuir	[65]
5	chitosan-activated carbon composite	MO dye	35.64 mg/g	PSO	Freundlich	[66]
6	CTA-CSM	MO dye	131.9 mg/g, 98.8%	PSO	Langmuir	[67]
7	CS-PSSS	MB dye	854 mg/g	PSO	Langmuir	[68]
8	CS	Cr(VI)	945.2 mg/g	PSO		[69]
9	CS-MA-DETA	Pb(II), Cd(II)	239.2, 201.6 mg/g	PSO	Langmuir	[70]
10	CM with PHEMA brushes	Cu(II)	299 mg/g	PSO	Langmuir	[71]

CYCTS: carboxylated chitosan, DS: diclofenac sodium, BSCM: bismuth cobalt selenide, CM, CSM: chitosan microsphere, CR: Congo red, CCQM: quaternized chitosan@chitosan cationic microsphere, CTA: trimethyl ammonium chloride, PSSS: poly(sodium 4-styrene sulfonate, CS: chitosan, PHEMA: poly(hydroxyethyl methacrylate), MA: methyl acrylate, DETA: diethylenetriamine.

**Table 8:** Summary efficiency of photocatalytic spheres.

S. No	Polymer	Catalyst	Adsorbate	Degradation Efficiency (%)	Reaction Time (min)	Reference
1	Chitosan	Fe	MB	98.8	6	[77]
2	PDA-PVDF	Fe species	MO	~100	60	[78]
3	PVA/SA	SA-FeCl_3_	TC	90.5	60	[80]
4	Alg-HAP, Alg-m, Alg-mHAP	HAP, Fe_3_O_4_	MO	71.42, 79.41, 84.28	90	[81]
5	CA	BiOCl	RhB	100	75	[82]
6	Chitosan	ZnFeO_4_	CDM	~100	7	[84]
7	Chitosan	FeSO_4_	Strong purple coloration, aromatic compound	91.92, 70	105	[85]

PVDF: polyvinylidene fluoride, Alg: alginate, mHAP: magnetic hydroxyapatite, m: magnetite, CA: calcium alginate, CDM: chlordimeform insecticide.

## Data Availability

Not applicable.
